# Trauma-focused psychological interventions for psychosis: Meta-analytic evidence of differential effects on delusions and hallucinations

**DOI:** 10.1017/S0033291725103036

**Published:** 2026-01-09

**Authors:** Diamantis Toutountzidis, Emily Ricketts, Keith R. Laws

**Affiliations:** School of Health, Medicine and Life Sciences, University of Hertfordshire, UK

**Keywords:** CBT, child abuse, cognitive behavior therapy, EMDR, eye movement desensitization and reprocessing, Post-traumatic stress disorders, psychosis, psychotherapy, randomized controlled trials, RCT, schizophrenia, trauma-focused treatment

## Abstract

Childhood trauma is a well-established risk factor for the onset and persistence of psychotic symptoms. Consequently, trauma-focused interventions (TFIs) are increasingly incorporated into psychosis treatment, though their effectiveness in reducing hallucinations and delusions remains unclear. This systematic review and meta-analysis evaluated the effects of TFIs on psychosis-related outcomes in individuals with psychotic disorders or subclinical symptoms. Thirty-six studies (*N* = 1,384) were included, with 18 (*N* = 806) contributing to meta-analyses. Study quality and risk of bias were assessed using AXIS, Cochrane RoB2, and GRADE. Pre–post analyses showed small reductions in hallucinations (*g* = −0.37; adjusted *g* = −0.28; *K* = 15) and medium reductions in delusions (*g* = −0.55; *K* = 14), with younger participants benefiting more. In controlled trials, TFIs did not significantly reduce hallucinations at the end of treatment or follow-up (*g* = −0.12 and −0.01; both *K* = 7), whereas delusions showed significant reductions at both time points (*g* = −0.44 and *g* = −0.48; both *K* = 7). No significant effect on negative symptoms was observed at the end of trial (*g* = −0.02; *K* = 6), though a small improvement appeared at follow-up (*g* = −0.26; *K* = 6). TFIs produced small but significant reductions in PTSD symptoms at both time points (*K* = 6). No consistent effects were found for secondary outcomes: depression (*K* = 7), anxiety (*K* = 5), or quality of life (*K* = 3), though functioning improved at follow-up (*K* = 6). TFIs appear particularly effective in reducing delusions, but show limited benefit for hallucinations and other secondary outcomes. Further work is needed to design and test symptom-specific psychological interventions for distinct psychotic experiences.

## Introduction

Psychosis is a multifactorial syndrome shaped by the interaction of biological, psychological, environmental, and societal factors (Howes & Murray, 2014; Morgan & Gayer-Anderson, 2016; Read, van Os, Morrison, & Ross, 2005; van Os, Kenis, & Rutten, 2010; Vassos et al. 2012). Among these, exposure to psychological trauma has emerged as a consistent and significant risk factor (Thompson & Broome, 2020). Evidence from meta-analyses indicates that approximately one-third of psychosis cases may be attributable to childhood adversity (Varese et al., 2012). A recent large-scale meta-analysis spanning four decades of research confirmed a strong association between childhood adversity and psychosis, with an overall odds ratio of 2.80 and particularly strong effects for emotional abuse (OR = 3.54), underscoring the clinical importance of early adversity in psychosis risk (Zhou et al., 2025). This association has also been highlighted in subclinical psychosis populations, where emotional abuse has emerged as one of the strongest predictors of psychosis-like experiences (Toutountzidis et al., 2022). Epidemiological studies show high prevalence rates of childhood abuse among individuals with schizophrenia: 26% report childhood sexual abuse, 39% physical abuse, and 34% emotional abuse (Bonoldi et al., 2013). These findings underscore the importance of trauma exposure as a potentially modifiable factor in the etiology of psychosis and raise questions about underlying mechanisms and therapeutic opportunities.

Post-traumatic stress disorder (PTSD) is a hypothesized pathway through which trauma may contribute to psychosis. PTSD affects around 16% of trauma-exposed youth (Alisic et al., 2014), and its persistence has been linked to heightened risk of psychotic experiences (Martin et al., 2023). Longitudinal studies suggest PTSD partially mediates the association between childhood trauma and psychotic-like experiences in adolescence (14%) and, to a lesser extent, in adulthood (8%) (Strelchuk et al., 2022). These findings highlight the importance of timely interventions targeting trauma-related distress to potentially interrupt the progression to more severe psychopathology.

Several neurobiological and psychological mechanisms have been proposed to explain how trauma confers risk for psychosis. Trauma exposure may lead to dysregulation of the hypothalamic–pituitary–adrenal (HPA) axis, heightening stress sensitivity (Walker, Mittal, & Tessner, 2008). Intrusions of trauma-related memory content may underlie certain hallucinatory experiences (Gracie et al., 2007), and early adversity can contribute to the development of negative core beliefs, thereby increasing vulnerability to persecutory delusions (Sitko et al., 2014). Emotional abuse has been identified as a reliable predictor of psychotic symptoms, potentially through its impact on self-concept (Toutountzidis et al., 2022). Moreover, trauma-related negative cognitions are common among individuals at high risk of psychosis (Morrison et al., 2006), suggesting that addressing these beliefs may be a useful therapeutic target (Ackner, Skeate, Patterson, & Neale, 2013; Zarubin, Gupta, & Mittal, 2023).

Given these associations, trauma-focused interventions (TFIs) have been increasingly applied in psychosis populations, particularly for comorbid PTSD (Sin et al., 2017). For this review, TFIs are defined as treatments that directly encourage a person face the memories, situations, and unhelpful thoughts or beliefs related to a traumatic experience, by using cognitive, emotional, or behavioral techniques to facilitate the processing of the experience (Schnurr, 2017; Wade et al., 2016). Evidence-based TFIs include trauma-focused cognitive behavioral therapy (TF-CBT) and eye movement desensitization and reprocessing (EMDR), both of which are recommended in clinical guidelines for PTSD (NICE, 2018). These interventions use exposure to the traumatic memory but differ in their mechanisms: exposure-based treatments, such as prolonged exposure (PE), aim to reduce avoidance and desensitize trauma responses, EMDR targets maladaptive memory networks through bilateral stimulation, while TF-CBT combines exposure with cognitive restructuring (Reid et al., 2023).

A meta-analysis by Brand, McEnery, Rossell, Bendall, and Thomas (2018) using pre–post analyses indicated a significant small pre–post treatment effect on positive symptoms (*K* = 7) and delusions (*K* = 4), but not for hallucinations (*K* = 4) or negative symptoms (*K* = 4). Pre–post treatment and follow-up data suggested that TFIs showed promising effects on reducing positive symptoms of psychosis at post-treatment (*g* = 0.31); however, these effects were small and not maintained at follow-up (*g* = 0.18). TFIs had a small effect on delusions at post-treatment (*g* = 0.37) and follow-up, but this was only significant at follow-up (*g* = 0.38). Crucially, Brand et al.’s (2018) review found no significant benefit specifically for hallucinations either at the end of treatment (*g* = 0.14) or at follow-up (*g* = −0.06). These findings are intriguing, considering much research in this area has proposed a relationship between hallucinations and traumatic events, with some suggesting they may be direct manifestations of trauma-based memories (Steel, 2015). Treatment length was found to significantly moderate both positive and negative symptoms at follow-up, suggesting this could be an important point of focus (Brand et al., 2018). However, an up-to-date synthesis of studies is needed as several new studies (including randomized controlled trials, RCTs) have been published since Brand et al.’s (2018) meta-analysis of hallucinations and delusions (in four pre–post studies and two RCTs). Additionally, most of the studies included in Brand et al.’s review were rated at high risk of bias and with low methodological quality. Only people with clinical presentations of psychosis were included, and thus, the question remains whether TFIs could be used to alleviate subclinical symptoms of psychosis and prevent escalation into psychotic disorders.

The most recent systematic review of 17 studies in this area conducted by Reid et al. (2023) found that psychotherapies using exposure such as PE, EMDR, and TF-CBT were more likely to improve at least one symptom of psychosis than trauma-informed interventions (e.g., cognitive restructuring) that did not include exposure. Nevertheless, the review by Reid et al. did not include a meta-analysis, which reduces the precision and certainty of results. Additionally, Reid et al. (2023) included only four controlled studies, and the remaining were case series with small sample sizes, making it hard to generalize results. Like Brand et al. (2018), studies posed a variety of methodological issues – for example, lack of blinding of participants, risk of attrition bias, and unrepresentative samples. As Reid et al. (2023) did not synthesize data using meta-analyses, it was also unclear whether any intervention (e.g., those that involved exposure) had significantly reduced any symptoms of psychosis. Our review focused on examining hallucinations, delusions, and total negative symptoms separately as Brand et al. (2018) found TFIs impacted symptoms differently.

Much of the research in both Brand et al. (2018) and Reid et al. (2023) reviews also focused on treating PTSD, with symptoms of psychosis as a secondary outcome; interventions therefore were often not targeted toward traumatic memories that might be directly relevant to symptoms of psychosis, an important consideration as research has shown relationships between the content of psychosis experiences and traumatic events (see Vila-Badia et al., 2021).

The present systematic review and meta-analysis provides an updated quantitative synthesis of the effects of TFIs on psychosis symptoms, examining hallucinations, delusions, and negative symptoms separately. We investigated psychosis symptoms across the continuum, as such experiences are not confined to clinical diagnoses (van Os et al., 2009), and examining both clinical and subclinical presentations may inform the development of more effective preventative strategies.

## Methods

### Search strategy

**Figure 1.**
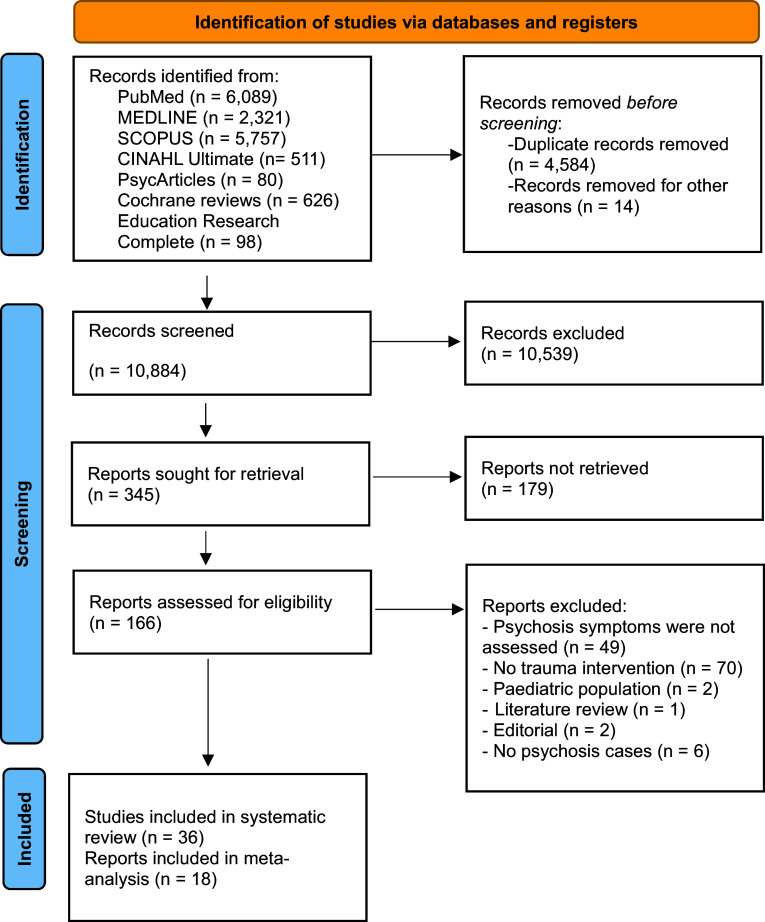
PRISMA 2020-compliant flow diagram of each stage and details of excluded reports in full review.

The review was preregistered with PROSPERO (CRD42024508790) and followed PRISMA 2020 guidelines for reporting systematic reviews and meta-analyses (see Figure 1 for flowchart and Supplementary 1 for the PRISMA 2020 Checklist (Haddaway & McGuinness, 2020)).

Literature searches were conducted on PubMed (*n* = 6,089), CINAHL Ultimate (*n* = 511), PsycArticles (*n* = 80), Cochrane reviews (*n* = 626), Education Research Complete (*n* = 98), and MEDLINE (*n* = 2,321) using the following sets of search terms:

(Psychosis *OR* psychotic *OR* schizoty* *OR* psychosis-like *OR* psychotic-like *OR* subclinical psychosis *OR* schizophrenia *OR* attenuated psychotic symptoms *OR* hallucinations *OR* delusions *OR* magical ideation *OR* suspiciousness *OR* delusional ideation *OR* odd belie* *OR* eccentric behavi* *OR* odd speech *OR* constricted affect *OR* unusual perceptual experiences *OR* ideas of reference *OR* paranoia ideation) *AND* (Post-traumatic *OR* trauma *OR* stressful events *OR* traumatic incident) *AND* (therapy *OR* psychotherapy *OR* intervention *OR* early intervention trauma focused *OR* CBT *OR* cognitive therapy *OR* cognitive processing therapy *OR* exposure therapy *OR* EMDR *OR* eye movement desensitization *OR* narrative exposure therapy *OR* brief eclectic psychotherapy *OR* virtual reality exposure).

A broader search strategy was used for Scopus – as only one article came up compared to 6,089 on PubMed using the original strategy – and revealed 5,757 research articles in English:

(psychosis OR schizoty* OR psychosis-like OR subclinical OR schizophren*) AND TITLE-ABS-KEY (post-traumatic OR trauma* OR abuse OR maltreatment OR neglect OR victimisation) AND TITLE-ABS-KEY (psychotherapy OR emdr OR intervention OR behavioural OR dialogical OR behavioral OR narrative OR exposure OR virtual).

Studies published in these databases since their day of inception to screening were screened for inclusion. Searches were updated in mid-June 2025. Identified articles were uploaded to Covidence (*n* = 15,482). A total of 4,584 studies were removed as duplicates and 14 for other reasons (e.g., not in English, conference abstracts). We screened 10,884 study titles manually using Covidence and classified 345 articles as potentially relevant. Following title and abstract screening, 166 articles were identified for full-text screening, and finally, after excluding 130 articles (See Supplementary 2 for full list), 36 studies were included in the systematic review. Out of these 36 studies, some were identified as follow-up investigations that utilized the same participant sample. For example, specific studies by van den Berg et al. (2015, 2016, 2018), de Bont et al. (2016), and Burger et al. (2025) all reported on a shared sample. In this case, we further examined de Bont et al. (2016) as this study presented data on hallucinations and delusions for two TFIs. For the meta-analyses, sufficient data were obtained from 18 studies that fulfilled the inclusion criteria. We approached researchers to request the data from studies where outcomes of psychosis symptoms were not available in the published papers. We received additional datasets for papers by Mueser et al., 2008 and Mueser et al., 2015.

### Eligibility criteria

To be included in the systematic review, studies had to meet the following criteria: (a) adult participants diagnosed with psychosis or symptoms of psychosis (i.e., clinical cases of a psychotic disorder or schizophrenia spectrum disorder or scoring highly for psychosis symptoms; or subclinical cases of those scoring highly on psychometric assessment of schizotypal traits or whose personality traits bare similarity to psychosis symptoms in early intervention and protective services); (b) studies that included TFIs for PTSD or post-traumatic stress symptoms; and (c) studies written in English. Screening and eligibility assessment were performed independently by two reviewers (DT and ER), and any disagreements were resolved by consensus.

### Data collection process and data items

The following data were extracted from each study: 1) study design, 2) participant characteristics (mean age and gender composition), 3) intervention and comparison groups, 4) primary outcomes, 5) secondary outcomes, and 6) treatment retention. A summary of all studies and their characteristics is presented in Table 1.

**Table 1.** Study characteristics

Study and design	Sample (N, age mean (SD), gender)	Clinical presentation and setting	TFI and dose	Psychosis outcomes (pre, post, follow-up means)	Attrition	AXIS quality score (max = 20)
Airey et al. (2023) – case series	*N* = 12, age 37.67 (12.75), female 5, male 7	Clinical diagnoses of SZ and other psychotic disorders. Outpatient Community Mental Health Team and Early Intervention Services NHS Trust, NW England	Adapted version of Taylor, Bee, Kelly, and Haddock’s (2019) attachment-focused iMAPS intervention. Mean number of sessions: 5.5	R-GPTS Persecutory subscale pre 17.5; post 13.5 Reference subscale pre 11.75; post 13.5	33% 2 during baseline, 2 during treatment	17
Arens (2015) – case study	*N* = 1, 45-yr-old male	PTSD and hallucinations. Recruited from an outpatient service in the USA	Trauma management therapy (Turner, Beidel, & Frueh, 2005) Length of intervention: 3 weeks	Number of self-reported weekly auditory hallucinations pre 7; post 1; 3-month follow-up:1 Number of self-reported weekly visual hallucinations pre 2; post 2; 3-month follow-up 0	0% No dropouts	12
Brand and Loewenstein (2014) – case series	*N* = 237, age and gender not reported	Dissociative identity disorder (criteria not reported). Recruited from many outpatient services in 19 countries From North America, Europe, South America, and Asia	Phasic trauma treatment for DID. Length not reported	SCL–90-R hearing voices item pre 1.89; 6-month follow-up 1.63; 18-month follow-up 1.49; 30-month follow-up 1.39	Total = 51% Time 2, *n* = 171 (25%); time 3, *n* = 131 (42%); time 4, *n* = 111 (51%)	11
Brand et al. (2020) – case studies	*N* = 2, case 1 late 30s, case 2 mid–40s, both female	Case 1 met criteria for borderline personality disorder and PTSD. Case 2 met criteria for major depressive disorder with psychotic features and PTSD	Trauma-focused imaginal exposure (Foa et al., 2007) Case 1: 6/6 sessions Case 2: 2/6 sessions	PSYRATS-AHS Case 1 pre 37; post 0; 1-month follow-up 0 Case 2 pre 34; post 39; 1-month follow-up 35	50% Case 2 dropped out after 2 sessions	15
Brand et al. (2021) – case series	*N* = 15, age 43.79 (8.64), female 9, male 5, other 1	Current auditory hallucinations (item K6b of MINI 7.02 Psychotic Disorders version) and a history of PTSD. Recruited from specialist voices clinic and auditory hallucinations research participant registry	Trauma-focused imaginal exposure (Foa et al., 2007) Length of intervention: 6 weekly 90-minute sessions	PSYRATS-AHS pre 29.58; post 26.08; 1-month follow-up: 21.08 PSYRATS-D pre 5; post 0; 1-month follow-up: 0	27% 1 dropout after baseline assessment. Of 14 who started therapy, 11 completed all 6 sessions	15
Buck et al. (2019) – RCT	*N* = 162, age 30.27(6.66), female 6, male 156	Diagnosis of PTSD on the Clinician-Administered PTSD Scale for DSM-IV Participants were active US army soldiers. Those with a diagnosis of past or current SZ, bipolar disorder, or psychotic disorder excluded	Prolonged exposure (PE) (Foa et al., 2007) versus 5-week waiting list control condition. Length: ten 90–120-minute sessions	BASIS–24 Scale – Psychosis item (Cameron et al., 2007); Significant reduction in persecutory ideation across both TF intervention and WL groups, with a significantly greater decrease over time in the TF condition. Auditory/visual hallucinations reduced from pre- to post-treatment; however, these were not exclusive to the TF intervention	31.7% dropout in those reporting hallucinations at baseline vs 33.7% of those who did not	15
Burger et al. (2025) – RCT	*N* = 39, age 36.85 (10.13), female 24, male 15, same sample taken from van den Berg et al. (2015)	Psychotic disorder on the Mini-International Neuropsychiatric Interview Recruited from an outpatient service in Netherlands	Participants were randomly allocated to 8 weekly sessions of PE or EMDR therapy, or to the WL condition	PSYRATS AHRS – Distress subscale TFT pre 15.10; post 14.40 WL pre 15.22; post 14.71 PSYRATS AHRS – Frequency subscale TFT pre 8.14; post 7.33 WL pre 8.44; post 7.43	25.6% dropouts	16
Callcott et al. (2004) – case study	*N* = 1, 34-yr-old, female	SZ and PTSD (ICD–10). Recruited from an outpatient service in the UK	Trauma-focused CBT (exposure, imagery rescripting, cognitive restructuring) Length: 17 sessions	Scale for the Assessment of Negative Symptoms (SANS) pre 8; post 4	0%	12
Cherestal and Herts (2021) – case study	*N* = 1, mid–40s, female	Diagnoses of PTSD and psychotic disorder not otherwise specified	PE (Foa et al., 2007) Length: 18 sessions	PSYRATS-AHS pre 29; mid 27; post 24	0%	11
de Bont et al. (2013) – case series	*N* = 10, age range 26–56, female 8, male 2	Diagnoses of PTSD (DSM-IV) plus a psychotic disorder. Recruited from a local Dutch mental health outpatient center	PE (Foa et al., 2007) or EMDR (Shapiro, 2001) Length: twelve 90-minute sessions	PSYRATS-AHS pre 14.54; post 10.67 PSYRATS-D pre 5.68; post 1.49	20% EMDR: 1/5 PE: 1/5	12
de Bont et al. (2016) – RCT	*N* = 155, age 41.2 (10.5), female 84, male 71, same sample with van den Berg et al. (2015)	Recruited from 13 Dutch psychosis treatment outpatient services (MINI plus criteria; 61.3% SZ, 29% SZA) plus chronic PTSD (DSM-IV)	PE vs EMDR vs waiting list Mean completed sessions: 7.1 in PE and 7.8 in EMDR Length: 8 weeks	GPTS PE pre 88.8; post 67.3; 6-month follow-up 65 EMDR pre 82.7; post 68; 6-month follow-up 70.2 WL pre 83.8; post 82.7; 6-month follow-up 78.3 Auditory Hallucinations Rating Scale PE pre 10.64; post 18.8; 6-month follow-up 22.5 EMDR pre 12.04; post 16.8; 6-month follow-up 16.1 WL pre 10.26; post 24.2; 6-month follow-up 16.8 Number in remission from psychotic disorders (SCI-SR-PANSS) PE (*n* = 53) pre 25 (47.2%); post 28 (59.6%); 6-month follow-up 18 (40.0%) EMDR (*n* = 55) pre 25 (45.5%); post 5 (56.8%); 6-month follow-up: 24 (55.8%) WL (*n* = 47) pre 19 (40.4%); post 12(30.8%); 6-month follow-up 18 (45%)	13 (24.5%) dropouts in PE 11 (20.0%) dropouts in EMDR	18
Every-Palmer et al. (2024) – RCT	*N* = 24, age 39.5 (11.5), female 8, male 16	Diagnoses of psychotic disorders or mood disorders with psychotic features (ICD–10). Inpatient, custodial, and community patients recruited under Regional New Zealand forensic service	EMDR (Shapiro, 2001) vs TAU Length: 9 sessions over 10 weeks	PSYRATS-AHS EMDR pre 8.8; post 3.3 WL pre 4.2; post 2.6 PSYRATS-D EMDR pre 3.8; post 1.3 WL pre 4.2; post 2.6	8.3% 2 dropped out from EMDR	14
Granier and Brunel (2022) – case study	*N* = 1, 35-yr-old, male	Diagnosis of schizophrenia (DSM-V). France	Singular EMDR session	PANSS total pre 113; 12-month post-treatment 35	None No further treatment needed after one session	11
Keen et al. (2017) – case series	*N* = 9, age 37 (11.34), female 4, male 5	All reported persecutory delusions and hallucinations. 5 (56%) had SZ (ICD–10), 2 (22%) had severe depressive episode with psychotic features. 2 (22%) had secondary PTSD diagnoses. Recruited from outpatient psychosis clinic	Integrated TF-CBTp protocol. Length: 9 months. Range: 8–35 months Median number of sessions: 41, range: 25–66	PSYRATS-D pre 13.57; post 8.33; 6-month follow-up 10.14 PSYRATS-AHS pre 29.56; post 20.50; 6-month follow-up 24.29	None 0%	14
Kim et al. (2010) – RCT	*N* = 45, age 32.6, female 33, male 12	Diagnoses of SZ (DSM-IV) and an inpatient stay of more than one week; recruited from an inpatient service in South Korea	EMDR vs progressive muscle relaxation vs TAU Length: 3 weeks	PANSS total EMDR pre 73.1; post 62.7; 2-yr follow-up 47.3 PMR pre 69.8; post 61.7; 2-yr follow-up 47.2 TAU pre 67.2; post 67.2; 2-yr follow-up 54.7 PANSS positive EMDR pre 16.9; post 12.2; 2-yr follow-up 10.0 PMR pre 15.9; post 12.9; 2-yr follow-up 9.0 TAU pre 18.8; post 15.4; 2-yr follow-up 11.6 PANSS negative EMDR pre 18.7; post 16.2; 2-yr follow-up 12.6 PMR pre 18.5; post 17.4; 2-yr follow-up 14.1 TAU pre 18.5; post 17.4; 2-yr follow-up 15.1	11% 5 dropouts	18
Marlow et al. (2024) – RCT	*N* = 36, age 42 for EMDR, 34.4 for TAU, female 14, male 10 in EMDR, female 6 male 6 in TAU	Participants with psychotic disorder who had reported a history of trauma (7 bipolar, 8 PTSD, 23 SZ, 4 SZA)	EMDR vs TAU Mean number of EMDR sessions: 4.6	PANSS total EMDR pre 69.7; post 61.3 TAU pre 68.0; post 65.3 PANSS positive EMDR pre 16.8; post 14.5 TAU pre 14.9; post 13.4 PANSS negative EMDR pre 14.9; post 13.8 TAU pre 14.8; post 16.3	Two randomized to EMDR did not commence treatment. No other dropouts during intervention. 9/36 participants lost to follow-up	17
McCartney et al. (2019) – case study	*N* = 1, 30-yr-old, female	First episode psychosis, diagnosed with psychotic disorder with social anxiety. Recruited from early intervention in psychosis service in the UK	TF-CBT (coping skills, imagery rescripting, exposure) Length: 22 sessions	PSYRATS-AHS pre 36; post 23	0% No dropouts	13
Mueser et al. (2008) – RCT	*N* = 108, age 44.21 (10.64), female 85 male 23 CBT group, *n* = 54, age 45.13 (9.83), female 41, male 13 TAU group, *n* = 54, age 43.30 (11.41), female 44, male 10	Primary diagnosis SZ; SZA; major depression; bipolar disorder secondary diagnoses BPD; substance use community mental health centers in the northeastern United States	CBT for PTSD program eight modules focused on information, crisis planning, psychoeducation, cognitive restructuring, and closure, and run for 12 to 16 sessions	BPRS (Lukoff, Nuechterlein, & Ventura, 1986) CBT pre 43.92; post 39.63; 3-month follow-up 40.57; 6-month follow-up 41.78 TAU pre 43.77; post 42.25; 3-month follow-up 43.97; 6-month follow-up 46.60	TI-CBT: received >5 sessions of CBT program (*n* = 44) analyzed after treatment (*n* = 32) 3 months post (*n* = 30) 6 months post (*n* = 33)	17
Mueser et al. (2015) – RCT	*N* = 201, 97 in brief group (female 65, male; 32 mean age 44.52); 104 in CBT group (female 73, male 31; mean age 42.96)	Diagnoses of SZ, SZA, major depression, or bipolar disorder (DSM-IV), plus diagnosis of severe PTSD (CAPS criteria). Recruited from 3 inpatient services and 2 outpatient services in the USA	Trauma-informed CBT vs brief treatment program (same breathing retraining and educational components as TF-CBT but without cognitive restructuring)	PANSS total TI-CBT pre 65.75; post 62.25 6-month follow-up 64.10; 12-month follow-up 60.21 Brief group pre 67.18; post 61.33; 6-month follow-up 65.37; 12-month follow-up 66.72	TI-CBT: 22/92 (24%) Brief treatment: 4/88 (5%)	17
Newman-Taylor et al. (2020) – case series	*N* = 15, age range 18–30, M = 20.67 (2.92), female 13, male 2	Participants scored high on non-clinical paranoia (42.7+ on the Paranoia Scale) and reported a recurrent traumatic memory	Single session of imagery rescripting	Paranoia Scale (Fenigstein & Vanable, 1992) pre 54; post 44.60	No dropouts	16
Paulik et al. (2019) – case series	*N* = 12, age range 20–62, M = 41 yrs, female 9, male 3	Nine participants had schizophrenia spectrum disorder. All were attending Perth Voices Clinic, Australia, to work on trauma and voices	Imagery rescripting Mean length: 11.75 weeks, range 9–19	PSYRATS-AH Distress pre 16; mid 13; post 12 PSYRATS-AH Severity pre 9; mid 7; post 6 Beliefs About Voices Questionnaire (Malevolence) pre 9; mid 9; post 8 Beliefs About Voices Questionnaire (Omnipotence) pre 11; mid 11; post 10	8.3% 1/12 dropped out	14
Quevedo et al. (2021) – case series	*N* = 6, age range 30–65, M = 45.2, female 4, male 2	Met criteria for current psychotic disorder, PTSD, and mild intellectual disability. Recruited from a tertiary mental health setting in the Netherlands	EMDR Length: 12 weekly sessions, 90 minutes each	Auditory Hallucinations Rating Scale pre 27; post 17; follow-up 15	0% No dropouts	16
Slotema et al. (2019) – case series	*N* = 47, age M = 37.4, female 41, male 6	Personality disorder (DSM-IV-TR) criteria plus PTSD. Recruited from an outpatient service in the Netherlands	EMDR vs TAU minimum: 2 median: 4 maximum: 15	PSYRATS-AHS no M and SD provided. Wilcoxon signed rank last observation carried forward (pre–post) significant decrease following treatment, *Z* = −2.12 (*p* = 0.034), Hedge’s *g* = 0.2	32% 15/47	15
Steel et al. (2017) – RCT	*N* = 61, age and gender not reported	Met criteria for psychotic disorder and PTSD. Recruited from two large NHS Trusts in South of England	Trauma-informed CBT (psychoeducation, cognitive restructuring) (Mueser et al., 2015) vs TAU length: 12–16 sessions, mean number of sessions: 12.3	PANSS positive TI-CBT pre 19.1; post 17.8 TAU pre 18.3; post 19.8 PANSS negative TI-CBT pre 16.3; post 15 TAU pre 15.3; post 16.4 PSYRATS-AHRS TI-CBT pre 16.9; post 16.8 TAU pre 16.4; post 14 PSYRATS-D TI-CBT pre 11.8; post 10 TAU pre 12.5; post 10.7	15% 4/27	17
Strous et al. (2005) – case series	*N* = 24, age 59–97, 72.2, female 10, Male 14	Participants were holocaust survivors diagnosed with SZ, recruited from 2 inpatient services in Israel	3-hour video testimony session over 2 sessions	PANSS total pre 68.6; post 69.8 PANSS positive pre 14.4; post 14.3 PANSS negative pre 29.9; post 23.9	0% No dropouts	16
Tong et al. (2017) – case series	*N* = 8, age 18–27, M = 21.25, 7 female, 1 male	Diagnosed with psychotic disorders. Recruited from a pilot trial of intervention to address trauma for young people with first episode psychosis in Australia	Trauma-informed CBT case management (safety, psychoeducation, timeline of experiences and development of PTSD, formulation)	BPRS (psychosis symptoms) pre 56.6; post 35.7	26.7% 4/15 dropouts following an invitation to take part	13
Trappler and Newville (2007) – case series	*N* = 24, age and gender not reported	Diagnoses of SZ or SZA (DSM-IV) plus PTSD (DSM-IV); recruited from 3 inpatient services in the USA	Group trauma-informed CBT (TICBT: emotion regulation and behavior/coping strategies to trauma triggers) vs supportive psychotherapy by therapists unfamiliar with trauma management. Length: 12 weeks	BPRS total significant decrease in TI-CBT group post-treatment: *z* = −4.20 (*p* < 0.001) BPRS subscale – hallucinatory behavior non-significant decrease in TI-CBT post-treatment BPRS subscale – unusual thought content – significant decrease in TI-CBT group post-treatment: *z* = −2 (*p* = 0.046) BPRS subscale – suspiciousness significant decrease in TI-CBT group post-treatment: *z* = −4.24 (*p* < 0.001)	Not reported	12
van den Berg and van der Gaag (2012) – case series	*N* = 27, age 45 (9.37), female12, male 15	Diagnosed with psychotic disorders and PTSD. Recruited from outpatient secondary mental health services in the Netherlands	EMDR Length: six weekly sessions for 90 minutes	GPTS (Green et al., 2008) pre 73.04; post 67.92	18.5% dropout rate	15
van den Berg et al. (2015) – RCT	*N* = 155, age 41.2 (10.5), female 84, male 71	Participants with lifetime psychotic disorder and current chronic PTSD. Recruited from 13 outpatient services in the Netherlands for severe mental illness	TF intervention (PE or EMDR) vs WL Length: 8 sessions	GPTS TF treatment pre 85.7 Symptom exacerbation baseline to post-treatment: 3/91 (3.3%) WL pre 83.8 Symptom exacerbation baseline to post-treatment: 2/39 (7.7%) Session rating of paranoid ideation Week before first TF session: 4.53 Week after first TF session: 3.85 Session rating of auditory verbal hallucinations Week before first TF session: 2.71 Week after first TF session: 2.62	24 treatment dropouts	17
van den Berg et al. (2016) – RCT	See van den Berg et al. (2015)	See van den Berg et al. (2015)	Treated PE and EMDR as one TF treatment vs WL and added adverse events and revictimization as measures. Length: 8 sessions	Patients in TF were significantly less likely to experience adverse events than participants in WL (OR = 0.48, *P* = .032, 95% CI [0.25, 0.94]). Patients in TF treatment were significantly less likely to experience revictimization than WL (OR = 0.40, *P* = .035, 95% CI [0.17, 0.94])	Dropout analysis was based on data of only 9 of the 24 treatment dropouts (the remaining participants missed the post-treatment assessment)	17
van den Berg et al. (2018) – RCT	85 participants at 12-month follow-up of van den Berg et al. (2015)	See van den Berg et al. (2015) as 12-month follow-up results to this study	See van den Berg et al. (2015) as 12-month follow-up results to this study	M or SD not provided. No significant changes in severity of paranoid thinking in PE or EMDR. No changes in auditory hallucinations in PE or EMDR or in hallucinations between 6 months and 12-month follow-up for PE or EMDR	The intent-to-treat sample comprised 155 participants at baseline, 130 at post-treatment, 128 at 6-month follow-up, and 85 participants at 12-month follow-up (only PE and EMDR conditions)	15
Varese et al. (2021) – case series	*N* = 19, age 16–45, 28.1 (9.6), female 11, male 8	Diagnosed with psychotic disorders. All had history of trauma. Recruited from community mental health teams and early intervention services in Northeast of England	CBTp modified to include therapeutic techniques suitable for trauma and dissociative experiences Length: 24 sessions	PSYRATS-AH pre 33.79; mid 29.00; post 23.64; 6-month follow-up 24.50 PSYRATS-D pre 16.44; mid 8.67; post 8.86; 6-month follow-up 5.83	15.8% 3 dropouts	13
Varese et al. (2024) – RCT	*N* = 60, age 17–62, 36.01 (13.15), female 36, male 24	Recruited from four early intervention services in the UK. All had a diagnosis of schizophrenia spectrum disorders or met local early intervention criteria defined using PANSS or CARMS. All reported at least 1 traumatic event on Trauma Screening Questionnaire (de Bont et al., 2015)	EMDRp + TAU vs TAU Average 10.8 EMDRp sessions (range 0–16, median 12)	PSYRATS-AHS EMDRp+TAU pre 18.5; 6-month follow-up 13.5; 12-month follow-up 16 TAU pre 16.1; 6-month follow-up 17.0; 12-month follow-up 13.2 PSYRATS-D EMDRp+TAU pre 15.8; 6-month follow-up 10.3; 12-month follow-up 7 TAU pre 13.6; 6-month follow-up 10.9; 12-month follow-up 7.8 PANSS EMDRp+TAU pre 66.8; 6-month follow-up 56.4; 12-month follow-up 50.7 TAU pre 67.9; 6-month follow-up 64.1; 12-month follow-up 54.4 GPTS EMDRp+TAU pre 91.0; 6-month follow-up 65; 12-month follow-up 61.4 TAU pre 83.9; 6-month follow-up 71.2; 12-month follow-up 60.4	4 dropouts following allocation to EMDRp+TAU. At 6 months, 5 lost to follow-up and 4 discontinued intervention. 10 lost to follow-up in TAU. At 12 months, 9 lost to follow-up in EMDRp+TAU and 9 lost to follow-up in TAU	14
Ward-Brown et al. (2018) – case series	*N* = 2, 20 and 17, both female	First-episode psychosis, recruited from early intervention in psychosis outpatient service in the UK	Case 1: TF-CBT and EMDR Length: 33 sessions Case 2: TF-CBT and EMDR Length: 32 sessions	PSYRATS-AHS Case 1: pre 33; post 29; 6-month follow-up 18 Case 2: pre 30; post 29; 6-month follow-up 20	No dropouts	12
Yasar et al. (2018) – case study	*N* = 1, 43 yrs, female	SZ diagnosis (criteria not reported). Recruited from an inpatient service in Turkey	2 sessions of EMDR over 2 weeks	PANSS total pre 78; post 34	No dropouts	12
Zhao et al. (2023) – RCT	*N* = 57, EMDR group, age 25.5 (4.3), WL group 24.6 (3.9), female 48, male 9	Diagnosed with PTSD and high risk of psychosis (score of 3–5 on one positive subscale at least in the SIPS). Recruited from an outpatient department in a tertiary psychiatric hospital in Beijing	EMDR vs WL Length: 90-min session weekly for 12 weeks	Total score of positive scales of the SIPS EMDR pre 10.0; post 5.0 WL pre 10.5; post 8.7	17.9% (5 dropouts)	18

*Note:* AHS, Auditory Hallucinations Subscale (of PSYRATS); BDI, Beck Depression Inventory; BAVQ, Beliefs about Voices Questionnaire; BPD, borderline personality disorder; BPRS, Brief Psychiatric Rating Scale; CAPS, Clinician-Administered PTSD Scale; CBT, cognitive behavioral therapy; CST, coping skills training; DES, Dissociative Experiences Scale; DID, dissociative identity disorder; DSM, Diagnostic and Statistical Manual of Mental Disorders; EMDR, eye movement desensitization and reprocessing; FU, follow-up; GAF, Global Assessment of Functioning; GPTS, Green Paranoid Thoughts Scale; HAM-D, Hamilton Depression Rating Scale; IR, imagery rescripting; M, mean; MDD, major depressive disorder; MINI, Mini-International Neuropsychiatric Interview; NR, not reported; PANSS, Positive and Negative Syndrome Scale; PD-NOS, psychotic disorder not otherwise specified; PE, prolonged exposure; PMR, progressive muscle relaxation; post, post-treatment; pre, pre-treatment; PSYRATS, Psychotic Symptom Rating Scales; PSYRATS-AHS, PSYRATS – Auditory Hallucinations Subscale; PSYRATS-D, PSYRATS – Delusions Subscale; PTSD, post-traumatic stress disorder; RCT, randomized controlled trial; S, session(s); SANS, Scale for the Assessment of Negative Symptoms; SCID, Structured Clinical Interview for DSM Disorders; SCL-90-R, Symptom Checklist-90-Revised; SD, standard deviation; SIPS, Structured Interview for Psychosis-Risk Syndromes; SZA, schizoaffective disorder; SZ, schizophrenia; TAU, treatment as usual; TF-CBT, trauma-focused cognitive behavioral therapy; TFT, trauma-focused treatment; TI-CBTp, trauma-informed cognitive behavioral therapy for psychosis; TSQ, Trauma Screening Questionnaire; WL, waitlist.

### Quality assessment tools

Study quality was assessed using the AXIS Appraisal Tool for Cross-Sectional Studies (Downes, Brennan, Williams, & Dean, 2016). The range of possible scores is from 0 to 20. The AXIS contains 20 items that assess: reporting quality (seven items: 1, 4, 10, 11, 12, 16, and 18), study design quality (seven items: 2, 3, 5, 8, 17, 19, and 20), and possible biases in the study (six items: 6, 7, 9, 13, 14, and 15). All items are scored 1 for ‘Yes’ and 0 for ‘No’, except for items 13 and 19, which are reverse-scored because a ‘Yes’ response indicates a potential source of bias rather than a quality feature.

For RCTs, risk of bias was assessed using the revised Cochrane Risk of Bias tool (RoB2: Sterne et al., 2019). RoB2 classifications are either ‘low’ risk of bias, ‘high’ risk of bias, or ‘some concerns’.

We also used the Grading of Recommendations Assessment, Development and Evaluation (GRADE) system to assess the quality of evidence for each outcome and give an overview of confidence in the effect sizes (Guyatt et al., 2008). GRADE ratings fall into four categories (high, moderate, low, and very low) based on risk of bias, consistency, directness, precision, and publication bias.

## Results

Our searches identified and included 14 randomized controlled trials (Buck et al., 2019; Burger et al., 2025; de Bont et al., 2016; Every-Palmer et al., 2024; Kim et al., 2010; Marlow et al., 2024; Mueser et al., 2008, 2015; Steel et al., 2017; van den Berg et al., 2015, 2016, 2018; Varese et al., 2024; Zhao et al., 2023); 15 case series studies (Airey, Berry, & Taylor, 2023; Brand et al., 2021; Brand & Loewenstein, 2014; de Bont, van Minnen, & de Jongh, 2013; Keen, Hunter, & Peters, 2017; Newman-Taylor, McSherry, & Stopa, 2020; Paulik, Steel, & Arntz, 2019; Quevedo, de Jongh, Bouwmeester, & Didden, 2021; Slotema et al., 2019; Strous et al., 2005; Tong, Simpson, Alvarez-Jimenez, & Bendall, 2017; Trappler & Newville, 2007; van den Berg & van der Gaag, 2012; Varese et al., 2021; Ward-Brown et al., 2018); and 7 case studies (Arens, 2015; Brand, Hardy, Bendall, & Thomas, 2020; Callcott, Standart, & Turkington, 2004; Cherestal & Herts, 2021; Granier & Brunel, 2022; McCartney et al., 2019; Yasar et al., 2018). These 36 studies comprised 1,384 participants; of these studies, 29 provided gender composition details (55.53% were female and 44.47% male), and 27 provided age data, leading to an overall mean age of 37.84 years (SD = 9.75; range 16–97). The studies emerged from a variety of countries, including United States (*K* = 9); Netherlands (*K* = 9); United Kingdom (*K* = 8); Australia (*K* = 3); China (*K* = 2); France (*K* = 1); Israel (*K* = 1); New Zealand (*K* = 1); South; Korea (*K* = 1); and Turkey (*K* = 1).

### Study quality

Although the 20 AXIS items are not equally weighted, the mean score for the 36 studies was 14.06 (SD =2.01). The lowest AXIS study quality rating was 10 out of 20 (Granier & Brunel, 2022), and the highest was 18 (Kim et al., 2010; Zhao et al., 2023). Following recent research (Antczak et al., 2020), we classified AXIS quality scores according to the number of ‘1’ scores for the 20 items for each study – so, studies achieving 80% ‘1’ scores indicated high quality; 60–80% indicated moderate quality; and < 60% indicated low quality. Thus, of 36 studies, 10 (27.78%) were rated as having high quality, 22 (61.11%) as moderate quality, and 4 (11.11%) as low quality.

### Trauma-focused interventions

This section includes the TFIs found in the identified studies. As differentiated by Reid et al. (2023) in line with the principles of Ehlers and Clark (2000), both TF-CBT and trauma-informed CBT recognize the impact of trauma on thoughts, feelings, and behaviors; however, TF-CBT combines these elements with exposure to process traumatic memories through desensitization and cognitive restructuring, and the trauma-informed CBT interventions in this review do not have an exposure component. Exposure involves systematic confrontation with a trauma-related memory (imaginal exposure) or reminders of trauma (*in vivo* exposure) with the aim of encouraging habituation over time and a reduction in trauma response (Bryant et al., 2023). This approach on its own is often referred to as PE (Foa, Hembree, & Rothbaum, 2007). In contrast, while EMDR also involves exposure to trauma-related imagery through visualization, the focus is less on reliving with the aim of habituation and more on using bilateral stimulation such as eye movements during visualization to stimulate reprocessing of traumatic memories (Shapiro, 2001).

### Synthesis of main findings

#### Eye movement desensitization reprocessing (EMDR)

Eleven studies examined EMDR, with nine reporting improvements in psychosis symptoms. Two RCTs (Marlow et al., 2024; Varese et al., 2024) found significant reductions in PANSS scores compared to treatment as usual, and another (Zhao et al., 2023) found positive effects in subclinical psychosis. Several case series reported reduced hallucinations, while one study showed complete remission following a single session. However, one RCT (Every-Palmer et al., 2024) found no significant differences in psychosis symptoms, though trauma symptoms improved. Overall, EMDR showed promising effects, especially for delusions, though controlled evidence remains limited.

#### Prolonged exposure

Four studies evaluated PE, including one RCT using virtual reality delivery (Buck et al., 2019), which found improvements in hallucinations over time, though not significantly different from waiting list. Case series showed mixed results, with some reporting temporary distress or symptom worsening and discontinuation of treatment (Brand et al., 2021). While PE may reduce psychosis symptoms in some cases, variability in outcomes and adverse responses deserve caution.

#### Eye movement desensitization reprocessing or prolonged exposure

Two studies directly compared EMDR and PE. A large RCT (de Bont et al., 2016) found both interventions significantly reduced paranoia, with PE effects sustaining longer. However, neither significantly reduced hallucinations. A smaller case series (de Bont et al., 2013) reported reduced PTSD symptoms, with no worsening of symptoms of psychosis. These findings suggest both EMDR and PE may benefit certain symptoms, especially paranoia, though effects on hallucinations remain unclear.

#### Trauma-focused cognitive behavioral therapy

Four uncontrolled case series investigated TF-CBT, most reporting improvements in hallucinations and delusions, maintained at follow-up in some cases (Keen et al., 2017; Varese et al., 2021). Other case reports also noted reductions in negative symptoms and dissociation. While these findings suggest TF-CBT may benefit different symptom domains, the lack of controlled trials limits conclusions.

#### Trauma-informed cognitive behavioral therapy

Four studies used trauma-informed CBT without exposure. Case series showed mostly positive outcomes, although temporary symptom worsening was reported (Tong et al., 2017). Two RCTs (Mueser et al., 2015; Steel et al., 2017) showed mixed results: both groups reduced PTSD symptoms, but psychosis symptoms did not differ significantly from control or improved more slowly. Trauma-informed CBT may be helpful, particularly when combined with preparatory or stabilizing work, though results were not consistent.

#### Other trauma-focused interventions

Six studies examined alternative trauma-focused approaches, including imagery rescripting (Newman-Taylor et al., 2020; Paulik et al., 2019), trauma management therapy (Arens, 2015), and phasic trauma treatment (Brand & Loewenstein, 2014). These generally reported positive effects on paranoia, hallucinations, and trauma-related distress. However, other approaches (e.g., iMAPS or trauma interviews alone) yielded limited or no effect on psychosis symptoms (Airey et al., 2023; Strous et al., 2005). The heterogeneity of methods and designs limits generalizability, though exploratory evidence suggests imagery-based approaches may be promising.

Overall, EMDR, TF-CBT, and PE were conceptualized as including exposure. All other TFIs examined (i.e., imagery rescripting, trauma-informed CBT, trauma management therapy, phasic trauma treatment, iMAPS, and trauma interviews) were categorized as non-exposure approaches, as they do not involve systematic or prolonged confrontation with trauma memories.

### Meta-analysis

A meta-analysis synthesis of studies was conducted by two authors (KRL and ER) using Comprehensive Meta-Analysis (CMA) 4.0. All final meta-analyses including meta-regression were completed by KRL. Primary outcomes were hallucinations and delusions, with negative symptoms of psychosis, PTSD, depression, anxiety, functioning, and quality of life as secondary outcomes. For between-group comparisons, we assessed effect sizes for the end of trial and follow-up. Both people with subclinical symptoms of psychosis (e.g., Brand & Loewenstein, 2014; Newman-Taylor et al., 2020) and clinical populations were included in meta-analyses. Random effect models were used for all analyses. Hedge’s *g* effect sizes were calculated for pre–post treatment effects across all studies. A correlation of 0.5 was assumed between pre- and post-analyses. For RCTs, we ran analyses comparing end-of-trial symptom scores for intervention and control groups. If studies did not provide means and standard deviations, effect sizes were calculated using r values and sample size (see van den Berg & van der Gaag, 2012). The *Q* and *I* ^2^ statistics were used to assess effect size heterogeneity.

For meta-regression and subgroup analyses, we followed the recommendations of no fewer than ten studies for a continuous variable and at least four studies per group for a categorical subgrouping variable (Fu et al., 2011). Following guidance from the Cochrane Handbook on Systematic Reviews (Cumpston et al., 2019), tests for funnel plot asymmetry were applied to analyses where at least ten studies were included in the meta-analysis (as fewer studies make the power of tests too low to distinguish chance from real asymmetry). Of the 36 studies included in the systematic review, 18 were included in the meta-analyses. Reasons for exclusion from meta-analyses included: case studies with small numbers, i.e., one or two participants (Brand et al., 2020; Callcott et al., 2004; Cherestal & Herts, 2021; Granier & Brunel, 2022; McCartney et al., 2019; Yasar et al., 2018), lacking data (Slotema et al., 2019; Trappler & Newville, 2007; Ward-Brown et al., 2018), not focusing on hallucinations, delusions, or negative symptoms (Tong et al., 2017), analyzing only pre–post data on negative symptoms (Strous et al., 2005), including only outcomes on positive symptoms (Zhao et al., 2023), using the same sample with other studies (Burger et al., 2025; van den Berg et al., 2015, 2016, 2018), or using unreliable or single-item outcome measures (Arens, 2015; Buck et al., 2019).

#### Hallucinations (pre–post)

**Figure 2.**
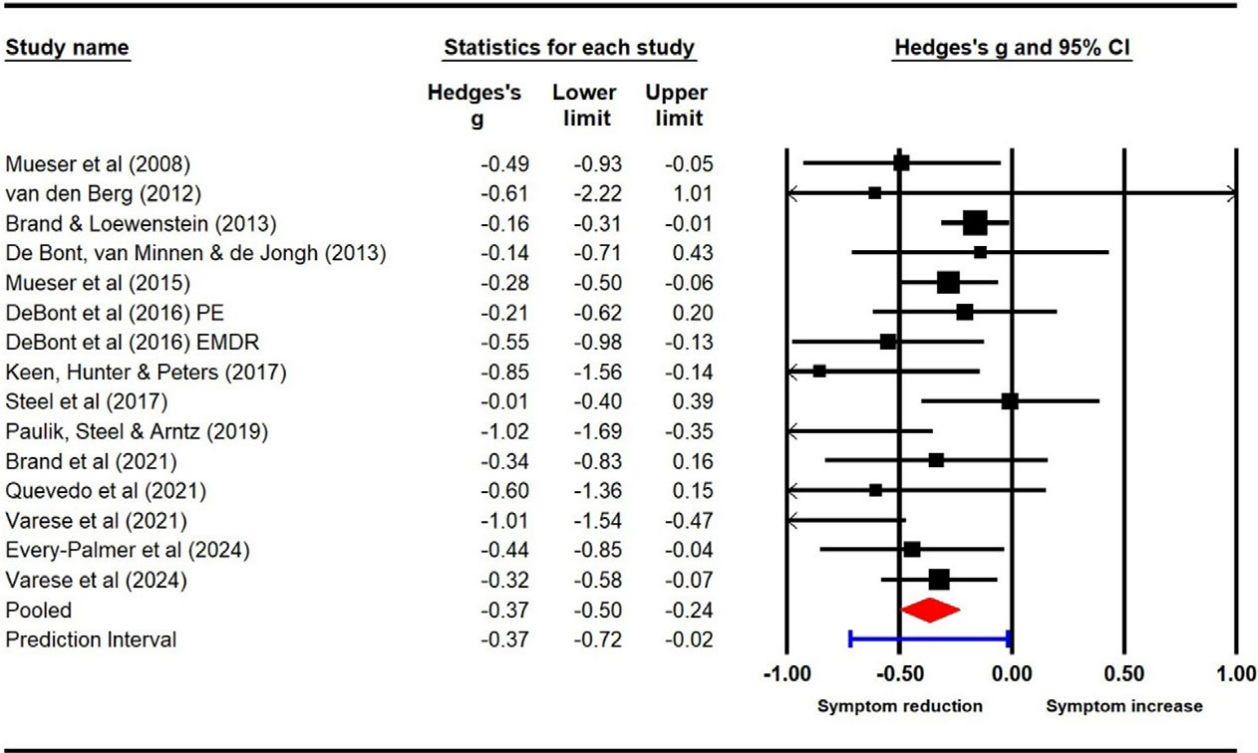
Pre–post analyses for hallucinations.

Fourteen studies (15 samples; *N* = 506) were included in the meta-analysis for hallucinations. Pre–post analyses indicated significant post-treatment effects for hallucinations (Hedge’s *g* = −0.37 [95% CI −0.50, −0.24]; prediction interval −0.72 to −0.02), suggesting a small–moderate reduction in reported symptoms of hallucinations following TFIs. The forest plot showing the within-groups analysis for hallucinations at post-treatment is presented in Figure 2. Heterogeneity was moderate (*Q* = 22.71, df = 14, *p* = .07; *I* ^2^ = 38.34). Trim-and-fill analysis suggested five potentially missing studies. The resulting adjusted effect size was reduced, but remained significant (*g* = −0.28; 95% CI −0.43 to −0.13).

Observation of the funnel plot revealed asymmetry and possible small-study effects (see Supplementary Appendices).

#### Hallucinations (between group) – end of trial and follow-up

**Figure 3.**
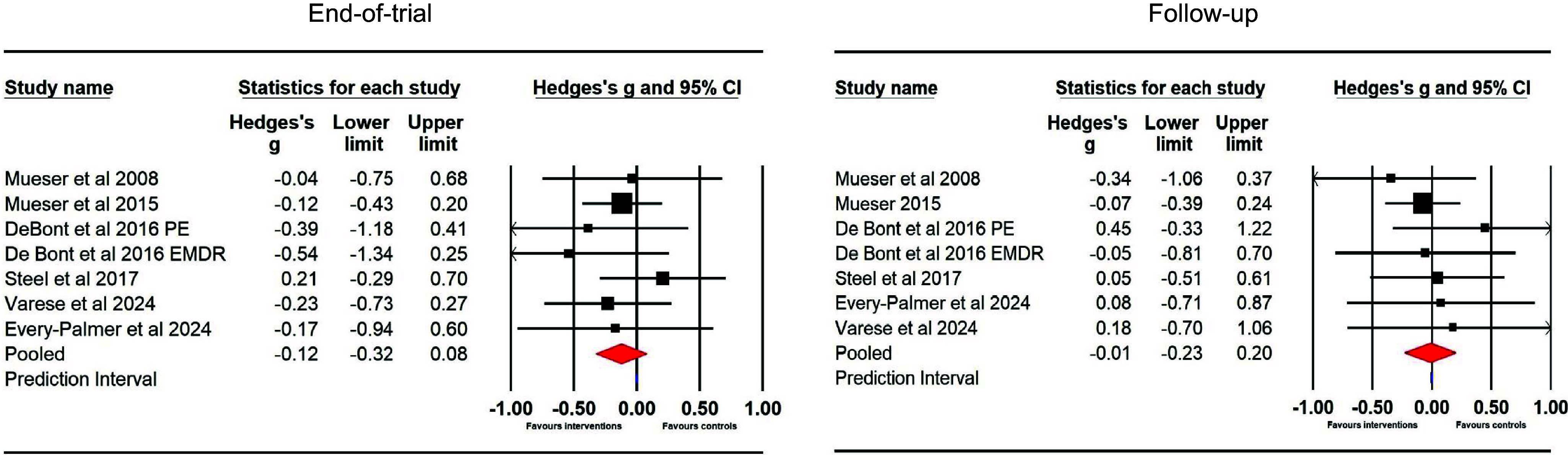
Hallucination ratings in between-group comparisons at the end of trial and follow-up.

Six studies (seven samples) used RCTs to compare TFIs to a control group at the end of trial (*N* = 222 intervention and *N* = 172 control) and at follow-up (*N* = 190 intervention and *N* = 147 control). The effect size for TFIs did not differ significantly from controls at the end of trial (*g* = −0.12 [95% CI −0.32 to 0.08]), with no heterogeneity (*Q* = 3.43, df = 6, *p* = .75; *I* ^2^ = 0); or at follow-up (*g* = −0.01 [95% CI −0.23 to 0.21]), with no heterogeneity (*Q* = 2.63, df = 6, *p* = .85; *I* ^2^ = 0; see Figure 3).

#### Delusions (pre–post)

**Figure 4.**
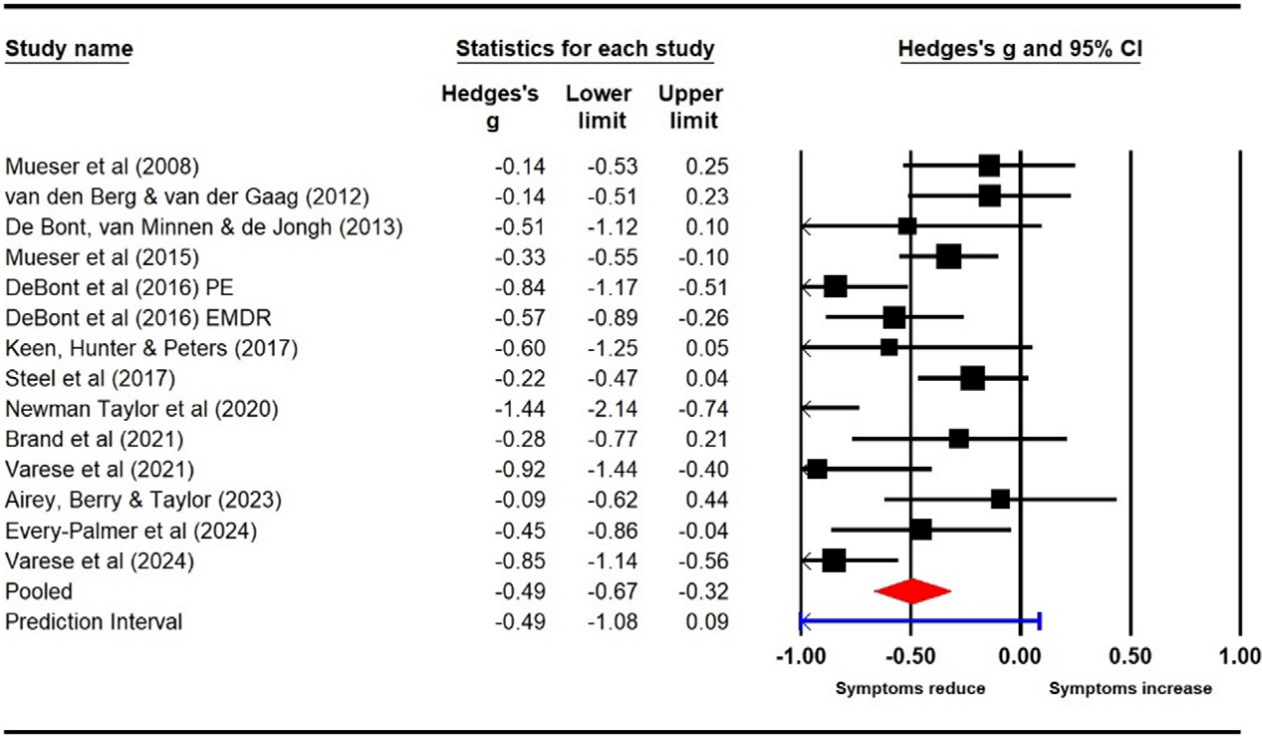
Pre–post analyses for delusions.

Thirteen studies (14 samples; *N* = 447) were included in the meta-analysis for delusions. Pre–post analyses using CMA indicated significant post-treatment effects for delusions (Hedge’s *g* = −0.49, 95% CI [−0.67, −0.32], *p* < .001) with a prediction interval of −1.08 to 0.09. The heterogeneity statistics suggested moderate heterogeneity (*Q* = 36.10, df = 13, *p* < .001; *I* ^2^ = 63.99). Overall, the findings indicate a reduction in the reported symptoms of delusions following TFIs with a medium effect size (see Figure 4).

The funnel plot did not indicate any asymmetry that might suggest small study effects and possible publication bias (see Supplementary Appendices).

#### Delusions (between-group) – end of trial and follow-up

**Figure 5.**
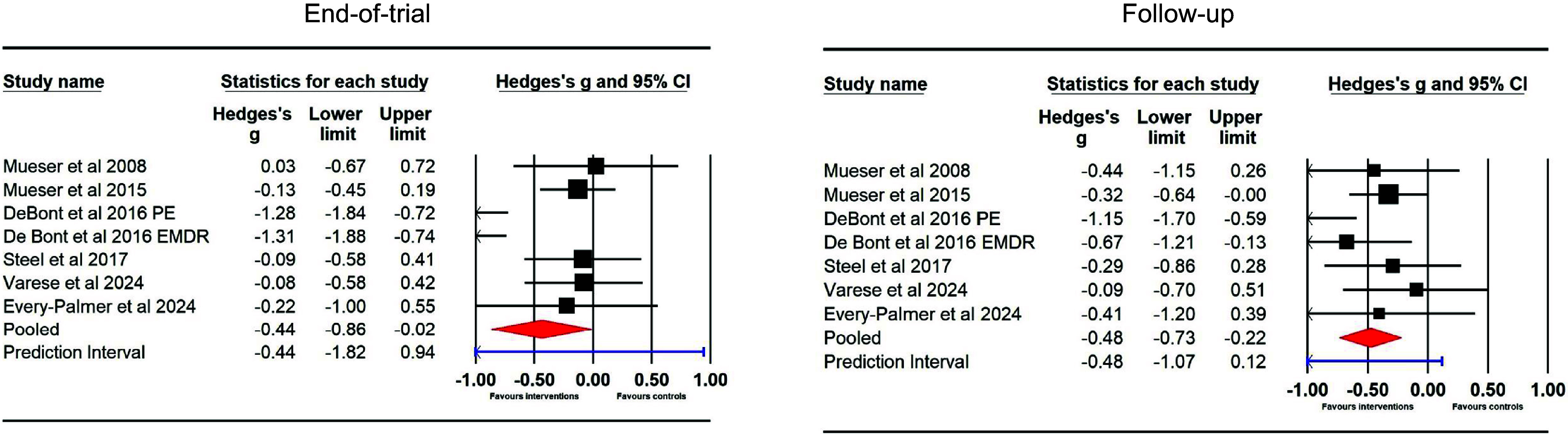
Delusions ratings in between-group comparisons at the end of trial and follow-up.

Six studies (seven samples) used RCTs to compare TFIs to a control group at the end of trial (*N* = 268 intervention and *N* = 194 control) and at follow-up (*N* = 249 intervention and *N* = 176 control). The effect size for TFIs differed significantly from controls at the end of trial (*g* = −0.44 [95% CI –0.86 to −0.02]), with high heterogeneity (*Q* = 26.74, df = 6, *p* < .001; *I* ^2^ = 77.56); prediction interval −1.82 to 0.94; and at follow-up (*g* = −0.48 [95% CI –0.73 to −0.22]); prediction interval −1.07 to 0.12; with moderate heterogeneity (*Q* = 8.91, df = 6, *p* = .18; *I* ^2^ = 32.68; see Figure 5).

#### Negative symptoms of psychosis (between-group) – end of trial and follow-up

**Figure 6.**
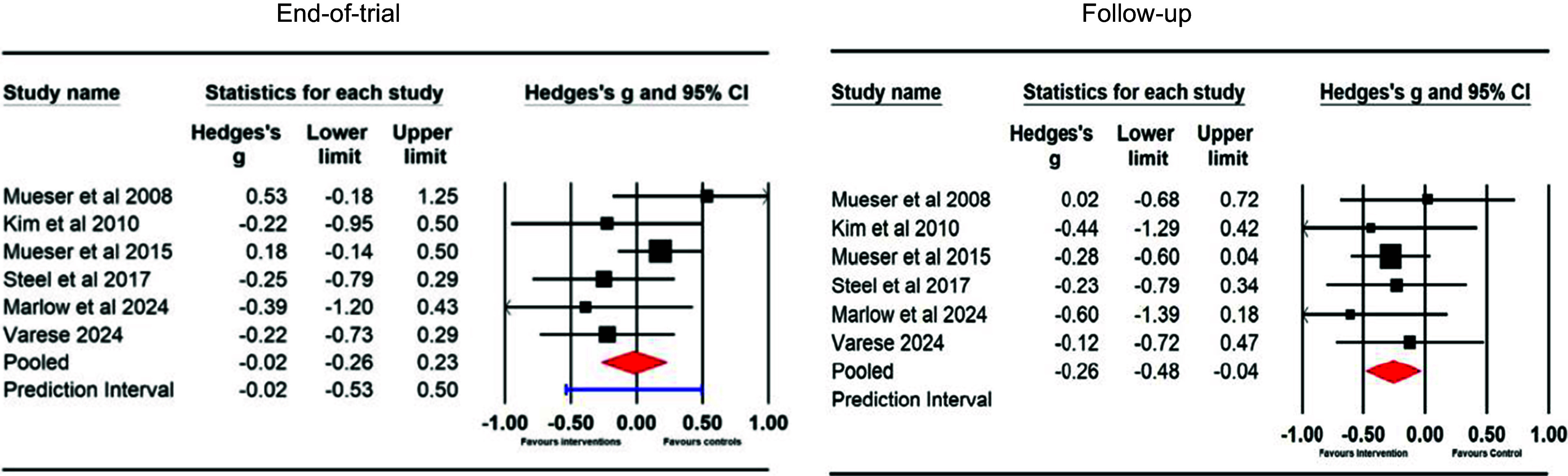
Negative symptoms of psychosis ratings in between-group comparisons at the end of trial and follow-up.

Six end-of-trial between-group comparisons (intervention *N* = 192; controls *N* = 158) were included in the meta-analysis of negative symptom outcomes. Analysis of these studies found no significant reduction in negative symptoms at the end of trial (*g* = −0.02; 95% CI [−0.26, 0.23], *p* = .89). The prediction interval was large at −0.53 to 0.50. Heterogeneity was moderate (*Q* = 6.17, df = 5, *p* = .29; *I* ^2^ = 19.01). At follow-up, analysis of these studies found a small but significant reduction in negative symptoms (*g* = −0.26; 95% CI [−0.48, −0.04], *p* = .02). Heterogeneity was low (*Q* = 1.75, df = 5, *p* = .88; *I* ^2^ = 0; see Figure 6). The forest plots did not show any asymmetry at either end of trial or follow-up.

### GRADE ratings

GRADE ratings indicate varying levels of confidence in the evidence for TFIs (see Supplementary Appendices). For uncontrolled pre–post studies, the evidence is rated as very low confidence for hallucinations and low for delusions – this partly reflects risk of bias from the lack of control groups, inconsistency in effect sizes, and imprecision from wide confidence intervals. Hallucinations also showed potential publication bias.

By contrast, evidence from between-group RCTs was rated moderate for hallucinations, delusions, and negative symptoms. Hallucinations produced consistent null findings with little or no heterogeneity. By contrast, controlled studies of delusions were downgraded for inconsistency, and negative symptoms were downgraded for imprecision. No clear evidence of publication bias was found for between-group outcomes. Overall, the strongest confidence in evidence exists for controlled trials of delusions, while evidence for any reduction of hallucinations and negative symptoms is minimal or less convincing.

### Moderator analyses

Moderator analyses were conducted on pre–post analyses for hallucinations and delusions as the number of controlled between-group studies was too few to derive reliable moderator analyses. Meta-regression analyses showed that the number of sessions, the proportion of female patients, and AXIS study quality did not predict effect sizes for hallucinations or delusions. In contrast, age was a highly significant predictor of effect size for delusions and marginally significant for hallucinations, with greater efficacy in studies with younger samples (See Table 2).

**Table 2.** Meta-regression analyses for pre–post hallucination and delusion effect sizes

	*K*	Range	Hallucinations	Delusions
Mean age	14	20.67–45.13	*Z* = 2.08, *p* = .037	*Z* = 4.07, *p* < .00001
Proportion of females	14	.33–.87	*Z* = −0.26, *p* = .79	*Z* = −0.81, *p* = .41
Number of therapy sessions	14	1–41	*Z* = −1.35, *p* = .18	*Z* = −0.30, *p* = .76
AXIS study quality	15	11–18	*Z* = −0.01, *p* = .99	*Z* = 0.79, *p* = .43

We used a subgroup analysis to compare effect sizes for hallucinations in pre–post studies using exposure (*g* = −0.35 [−0.47, −0.22]; *K* = 10) versus no exposure (*g* = −0.46 [−0.82, −0.11]; *K* = 5) and found no difference (*Q* = 0.37, df = 1, *p* = .54). A subgroup analysis was also used to compare delusion effect sizes for pre–post studies involving exposure (*g* = −0.56 [−0.76, −0.37]; *K* = 10) and no exposure (*g* = −0.31 [−0.62, −0.01]; *K* = 4) and again found no difference (*Q* = 1.81, df = 1, *p* = .18).

Further, several studies were identified as feasibility trials (Airey et al., 2023; Brand et al., 2021; de Bont et al., 2013; Keen et al., 2017; Kim et al., 2010; Quevedo et al., 2021; Slotema et al., 2019; van den Berg & van der Gaag, 2012; Varese et al., 2024; Zhao et al., 2023). An exploratory examination revealed no difference in pre–post effect sizes for delusions in feasibility (*g* = −.50 [−.72 to −.28; *K* = 9) versus other trials (*g* = −.48 [−.81 to −.16] *K* = 5): *Q* = .01, df = 1, *p* = .93). Similarly, for pre–post hallucination, effect sizes for feasibility (*g* = −.38 [−.56 to −.20; *K* = 9) and other trials did not differ (*g* = −.37 [−.56 to −.17] *K* = 6): *Q* = .01, df = 1, *p* = .92). Only one RCT (Varese et al., 2024) was confirmed as a feasibility trial, and so, we did not analyze RCTs alone.

### Secondary outcomes

Meta-analyses of between-group comparisons were conducted for PTSD, depression, anxiety, functioning, and quality of life at the end of treatment and follow-up. For PTSD symptoms (*K* = 6), small but significant effects favoring the intervention were observed at the end of treatment (*g* = −0.36, 95% CI −0.61 to −0.12, *I* ^2^ = 35%) and follow-up (*g* = −0.31, 95% CI −0.54 to −0.08, *I* ^2^ = 25%). For depression (*K* = 7), effects were small and not statistically significant at either time point: end of treatment (*g* = −0.24, 95% CI −0.51 to 0.02, *I* ^2^ = 58%) and follow-up (*g* = −0.11, 95% CI −0.36 to 0.13, *I* ^2^ = 50%). Similarly for anxiety symptoms (*K* = 5), no significant effects were found at the end of treatment (*g* = −0.16, 95% CI −0.43 to 0.11, *I* ^2^ = 46%) or follow-up (*g* = −0.17, 95% CI −0.36 to 0.01, *I* ^2^ = 0%). For functioning (*K* = 6), there was no effect at the end of treatment (*g* = 0.12, 95% CI −0.04 to 0.29, *I* ^2^ = 0), but a small, significant improvement emerged at follow-up (*g* = 0.28, 95% CI 0.11 to 0.45, *I* ^2^ = 0). For quality of life (*K* = 3), no significant effects were observed at either end of treatment (*g* = −0.13, 95% CI −0.37 to 0.10, *I* ^2^ = 0) or follow-up (*g* = −0.07, 95% CI −0.31 to 0.16, *I* ^2^ = 0).

### Risk of bias

Our searches identified 14 controlled trials, though four were reanalyses of the same trial and one used single-item outcome measures to capture psychosis-like experiences. We therefore conducted Cochrane RoB2 analyses of nine individual trials (see Supplementary Appendices). These showed that none of the included trials were at high risk of bias overall or in any individual domain.

## Discussion

This systematic review identified 36 studies (*N* = 1,384) evaluating the efficacy of TFIs for psychosis. Our meta-analyses provide updated evidence that TFIs consistently reduce delusions, with moderate effect sizes observed across both uncontrolled and controlled trials, including at follow-up. In contrast, hallucinations did not significantly improve in controlled trials, and although small-to-moderate improvements were seen in pre–post designs, these were attenuated after adjusting for publication bias. These findings indicate symptom-specific variability in treatment response, suggesting potentially distinct underlying mechanisms and a need for tailored intervention strategies.

Our analysis of RCTs revealed a dissociation in the effects of TFIs on delusions and hallucinations; with a significant and lasting reduction in delusions, but not for hallucinations. These findings reinforce and expand upon the previous meta-analysis of Brand et al. (2018), who similarly reported stronger TFI effects for delusions than for hallucinations. By tripling the number of studies included (*K* = 15 for delusions and *K* = 14 for hallucinations in pre–post analyses; *K* = 7 for each in controlled trials), we increase the statistical power and precision of this conclusion. Although effect sizes for delusions were comparable at the end of trial and follow-up, the prediction interval for follow-up was much smaller than that for the end of trial. These findings suggest that immediate treatment effects for delusions are quite variable across studies, while long-term effects are more stable and predictable. One clear implication is that the expected range of true effects for delusions appears more favorable at follow-up than at the end of trial, suggesting that TFIs produce a more stable and sustained reduction in delusional symptoms over time. This may reflect the challenging nature of TFIs, which can initially increase distress or exacerbate delusional thinking for some individuals.

In contrast to the findings for delusions, none of the RCTs conducted to date have reported a statistically significant reduction in hallucinations. It is important to recognize that several trials included relatively small samples, including one feasibility study (Varese et al., 2024), meaning that very small effects may not have been detectable. However, small sample size alone is unlikely to fully account for the pattern of results. The same seven RCTs that yielded no effect on hallucinations did detect moderate reductions in delusions at both end of trial and follow-up, indicating that the designs were capable of detecting effects of that magnitude. To examine this issue more formally, we calculated the minimum detectable effect size (MDES) for hallucination outcomes. Across RCTs, observed effects on hallucinations were small (*g* = −0.12 at end of trial; *g* = −0.01 at follow-up) and showed no between-study heterogeneity (*I* ^2^ = 0%). While MDES estimates indicate that trials were only powered to detect effects of approximately *g* ≥ 0.30 – so very small effects may have gone undetected **–** the consistently near-zero effect sizes suggest that any true impact of TFIs on hallucination outcomes is likely to be minimal. Together, these findings imply that TFIs may have limited influence on hallucinations, even if modest, sub-detectable effects cannot be ruled out.

While TFIs appear to impact the severity of delusions but not hallucinations, it remains possible that hallucination severity may be less appropriate than, for example, hallucination distress as a target for TFIs. Indeed, psychological therapy trials for psychosis have historically followed a pharmacological model of outcomes, placing primary emphasis on reducing positive symptoms (for discussion, see Birchwood & Trower, 2006; Laws et al., 2018). Similarly, most studies in the current review focused on symptom reduction, with few studies examining reductions in distress associated with such symptoms. Although only two studies in the current review (Brand et al., 2021; Paulik et al., 2019) assessed the impact of TFIs on distress associated with hallucinations, both reported large pre–post reductions in the distress associated with hallucinations. Future research should more explicitly prioritize patient-centered outcomes such as distress associated with hallucinations, as well as functioning and quality of life in sufficiently powered trials. These findings underline the importance of selecting outcomes that map onto the psychological processes TFIs are most likely to influence, particularly when assessing hallucinations.

This symptom-specific pattern of response raises important questions about the underlying mechanisms driving treatment effects. Hallucinations and delusions have both been associated with childhood trauma (Bailey et al., 2018), thus underpinning the rationale for TFIs in psychosis. Nonetheless, the differential treatment response seen here – in the same samples – points to possible differences in the underlying mechanisms of these two symptoms. Delusions, particularly paranoid or persecutory types, are often conceptualized as arising from maladaptive threat-based appraisals shaped by trauma (Freeman et al., 2002; Garety et al., 2001). In such cases, trauma-focused work may help individuals reconstruct more adaptive narratives, potentially diminishing the need for delusional explanations. By addressing maladaptive trauma-related schemas (e.g., beliefs about danger, trust, and self-worth), TFIs may effectively reduce the cognitive bias and threat perception that fuel delusional ideation (Brand et al., 2018; Hardy et al., 2005). While trauma exposure has been linked to the content and distress of hallucinations (Peach et al., 2021), the core phenomenology of hearing voices appears to be less responsive to change through both cognitive-based and exposure-based trauma interventions. Hallucinations – particularly auditory hallucinations – may differ because they are primarily associated with alterations in perceptual processing and underlying neural activity (Allen, Larøi, McGuire, & Aleman, 2008; Waters et al., 2012; Zmigrod, Garrison, Carr, & Simons, 2016). Indeed, hallucinations may stem from dissociative processes that persist independently of trauma meaning-making (Longden, Madill, & Waterman, 2012). So, although trauma may influence the content and emotional salience of hallucinations (Steel, 2015), TFIs may not directly address the perceptual anomalies or dissociative processes that give rise to the experience of hearing voices (see Frost, Collier, & Hardy, 2024). TFIs may therefore be well suited to altering trauma-related beliefs that fuel delusions, but have limited impact on the neurocognitive and perceptual mechanisms associated with hallucinations. These findings also suggest that collapsing delusions and hallucinations into a single ‘positive symptom’ outcome may obscure differential treatment effects and mechanisms (Steel et al., 2007).

Beyond symptom-specific mechanisms, individual characteristics may also shape differential response to TFIs. Our moderator analyses revealed that younger age significantly predicted larger pre–post TFI treatment effects for delusions and to a lesser extent also for hallucinations. This age-related effect may reflect multiple factors, including shorter illness duration, greater neurocognitive plasticity, or higher engagement among younger individuals. Younger people with psychosis are more likely to be in the early stages of illness, which has been associated with better responsiveness to psychological interventions (Stafford et al., 2015). Additionally, adolescents and young adults may retain greater neural flexibility, potentially enhancing their capacity to benefit from trauma-focused work (Paus, Keshavan, & Giedd, 2008). Additional planned moderator analyses of pre–post effect sizes for hallucinations and delusions showed that neither gender (proportion of female participants), number of therapy sessions, nor study quality significantly moderated treatment effects. Furthermore, and contrary to previous reports (Brand et al., 2018), we also did not find any higher efficacy for TFIs incorporating exposure techniques (e.g., EMDR, PE). However, this finding was based on pre–post analyses, and further research, especially using longitudinal or follow-up designs, is suggested to examine this result thoroughly.

Although the primary focus of this review was on positive symptoms, the differential pattern of effects also extended to negative symptoms. Although TFIs did not significantly reduce negative symptoms at the end of treatment, a small but significant improvement emerged at follow-up. This is a notable finding given the well-established difficulty of treating negative symptoms (Erhart, Marder, & Carpenter, 2006), though it should be interpreted cautiously. Negative symptoms were not a primary outcome in most included studies, and the review’s search strategy was not optimized for capturing them, raising the possibility that available data underestimate or incompletely reflect TFI effects. Moreover, small statistically significant improvements in negative symptoms – common across both pharmacological and psychological interventions – do not always translate into clinically meaningful change (see Fusar-Poli et al., 2015). Nonetheless, theoretical work suggests that aspects of negative symptomatology, such as emotional numbing, anhedonia, and social withdrawal, may overlap with trauma-related avoidance and emotional suppression (Brand et al., 2018; McGorry, 1991). TFIs that reduce avoidance and facilitate emotional processing could therefore plausibly influence negative symptoms over time. Future research should examine these potential shared mechanisms directly and assess whether TFIs can produce sustained and clinically relevant improvements in negative symptoms.

The symptom-specific pattern of response was broadly mirrored in secondary outcomes. We report small improvements in PTSD symptoms at both end of treatment and follow-up, as well as a modest enhancement in functioning at follow-up. However, TFIs did not significantly improve depression, anxiety, or quality of life outcomes at any time point. Nevertheless, whether such null findings represent a true lack of efficacy or not is hard to determine. Secondary outcomes have been assessed in only a small number of trials and crucially, not typically as a direct focus of TFIs and so may be both underpowered and insufficiently focused to detect such effects.

The current findings should also be considered in light of several methodological limitations that qualify the strength of the evidence. These methodological considerations are crucial for guiding the next generation of TFIs, which will need to be explicitly shaped around symptom-specific mechanisms and patient-centered outcomes. While pre–post designs permit meta-analyses of more studies and therefore enable moderator analyses, pre–post analyses are vulnerable to confounding influences such as spontaneous remission, regression to the mean, and placebo effects (Cuijpers, Weitz, Cristea, & Twisk, 2017). This limitation reinforces the importance of assessing controlled trials, which did confirm the moderate reduction for delusions, but not hallucinations. The confidence associated with evidence from pre–post studies (as rated using the GRADE) was low for delusions and very low for hallucinations. By contrast, all GRADE ratings for controlled studies of hallucinations and delusions at the end of trial and at follow-up revealed moderate confidence in the findings. Further, we note that on the Cochrane RoB2 assessment, none of the RCTs were deemed at high risk of bias, and all studies included in the review were rated as having moderate-to-high quality using the AXIS scale. In this context, the end of trial and follow-up analyses do provide reliable indicators of the true effects of TFIs for delusions and hallucinations.

## Conclusion

Together, these findings provide consistent evidence for a symptom-specific pattern of TFI response, with robust effects on delusions but less impact on hallucinations. This systematic review and meta-analysis indicates that TFIs are effective in reducing delusional symptoms in individuals with psychosis, with gains that are maintained over time. In contrast, TFIs showed limited efficacy for hallucinations, particularly in controlled trials at the end of trial or follow-up. Some benefit emerged in pre–post analyses, though they appeared to reflect the evidence of possible publication bias/small study effects. A significant reduction in negative symptoms at follow-up also emerged, although this outcome was not a central focus of the included studies and warrants further investigation. These findings suggest TFIs may be more effective for cognitive-affective processes underpinning delusions than for perceptual or dissociative mechanisms associated with hallucinations. Accordingly, future trials should treat hallucination distress – not severity – as a primary outcome when evaluating TFIs. These results expand upon earlier findings by including substantially more studies, especially RCTs, and offer the first reliable moderator analyses for delusions and hallucinations, albeit in pre–post analyses. The number of controlled trials nonetheless remains limited, and many studies did not directly address psychosis-related trauma, which may have influenced symptom-specific treatment outcomes. Researchers might also place greater emphasis on examining more patient-centered outcomes – such as functioning and quality of life – which would enhance clinical relevance. Future research should focus on (1) targeting trauma directly linked to symptoms of psychosis, (2) distinguishing between symptom domains when assessing treatment efficacy, and (3) identifying mechanisms and moderators that account for differential response. The evidence therefore points toward a future in which TFIs are developed and evaluated with explicit attention to symptom domain, underlying mechanism, and outcomes that matter to the patient.

## Supporting information

10.1017/S0033291725103036.sm001 Toutountzidis et al. supplementary material Toutountzidis et al. supplementary material

## Supplementary material

The supplementary material for this article can be found at http://doi.org/10.1017/S0033291725103036.
