# Supplementary material for: Trauma-focused psychological interventions for psychosis: Meta-analytic evidence of differential effects on delusions and hallucinations
Source: Psychol Med. 2026 Jan 9;56:e11. doi: 10.1017/S0033291725103036 (PMC12885351; doi:10.1017/S0033291725103036)
Supplement: Toutountzidis et al. supplementary material [file S0033291725103036sup001.zip › Appendices.docx]

**Appendices**

| **Funnel plot for pre-post hallucinations** |
| --- |
| 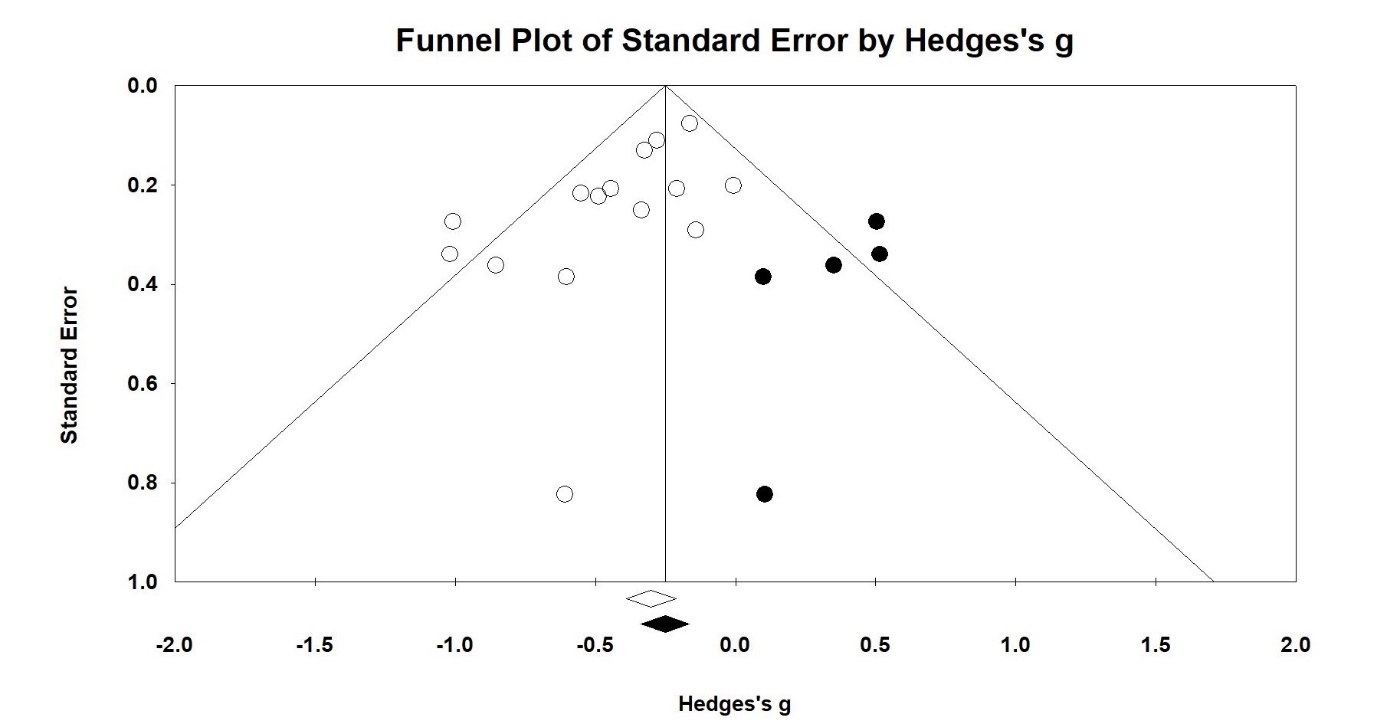 |

| **Funnel plot for pre-post delusions** |
| --- |
| 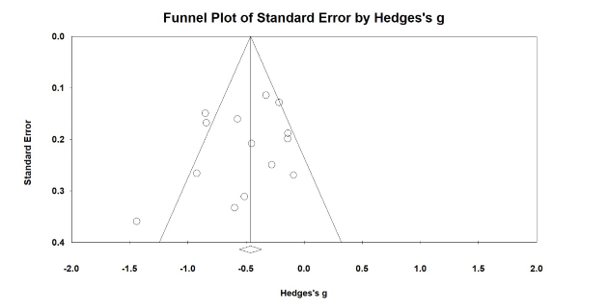 |

| GRADE Ratings | | | | | | | | |
| --- | --- | --- | --- | --- | --- | --- | --- | --- |
| Outcome & Analysis | Study Type | No. of Studies | Risk of Bias | Inconsistency | Indirectness | Imprecision | Publication Bias | Overall Certainty |
| Hallucinations (pre-post) | Pre-post | 14 studies | No control | Moderate (I² = 38%) | No concerns | Serious (95% CI includes very small effects) | Suspected (funnel plot asymmetry, 5 missing studies) | Very Low |
| Delusions (pre-post) | Pre-post | 13 studies | No control | Moderate (I² = 64%) | No concerns | Serious (wide prediction interval includes null/positive) | No concerns (no funnel plot asymmetry) | Low |
| Hallucinations (end-of-trial RCT) | RCT | 6 studies (7 samples) | No concerns | No concerns (I² = 0%) | No concerns | Serious (CI includes 0 and small effect) | too few studies for funnel | Moderate |
| Hallucinations (follow-up RCT) | RCT | 6 studies (7 samples) | No concerns | No concerns (I² = 0%) | No concerns | Serious (g = -0.01, wide CI) | too few studies for funnel | Moderate |
| Delusions (end-of-trial RCT) | RCT | 6 studies (7 samples) | No concerns | Serious (I² = 77%) | No concerns | No concerns | too few studies for funnel | Moderate |
| Delusions (follow-up RCT) | RCT | 6 studies (7 samples) | No concerns | Some concerns (I² = 32.7%) | No concerns | No concerns | too few studies for funnel | Moderate |
| Negative symptoms (end-of-trial RCT) | RCT | 6 studies | No concerns | No concerns (I² = 19%) | No concerns | Serious (null effect, wide CI) | too few studies for funnel | Moderate |
| Negative symptoms (follow-up RCT) | RCT | 6 studies | No concerns | No concerns (I² = 0%) | No concerns | Serious (CI narrowly significant; small sample) | too few studies for funnel | Moderate |

| **Cochrane Risk of Bias (RoB2) analyses** |
| --- |
| 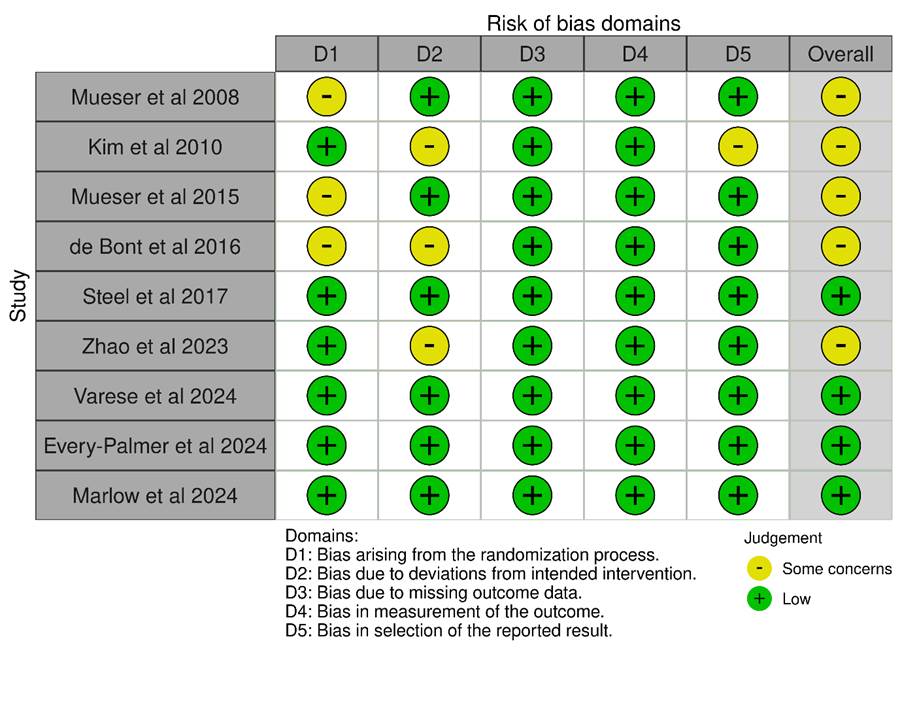 |
| 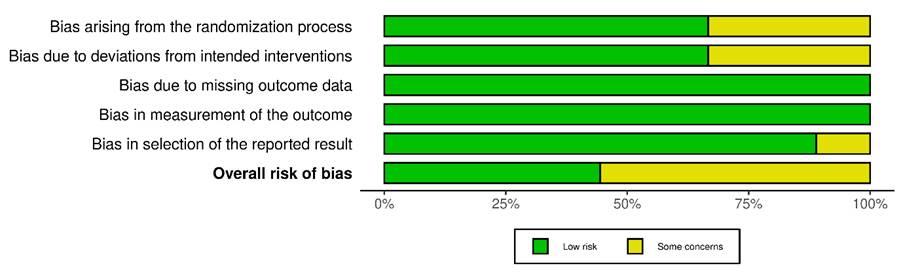 |
