# Supplementary material for: Trauma-focused psychological interventions for psychosis: Meta-analytic evidence of differential effects on delusions and hallucinations
Source: Psychol Med. 2026 Jan 9;56:e11. doi: 10.1017/S0033291725103036 (PMC12885351; doi:10.1017/S0033291725103036)
Supplement: Toutountzidis et al. supplementary material [file S0033291725103036sup001.zip › Suppl 2 - Exluded studies references list.docx]

**References of excluded studies**

Aas, M., Andreassen, O. A., Aminoff, S. R., Færden, A., Romm, K. L., Nesvåg, R., . . . Melle, I. (2016). A history of childhood trauma is associated with slower improvement rates: Findings from a one-year follow-up study of patients with a first-episode psychosis. *BMC Psychiatry, 16*(1), 126. doi:10.1186/s12888-016-0827-4

Addington, J., Van Mastrigt, S., & Addington, D. (2004). Duration of untreated psychosis: impact on 2-year outcome. *Psychological Medicine, 34*(2), 277-284. doi:10.1017/s0033291703001156

Agarkar, S. (2012). A case of prolonged duration of untreated psychosis: barriers to treatment and strategies to improve the outcome. *Clinical Schizophrenia & Related Psychoses, 6*(1), 45-48. doi:10.3371/CSRP.6.1.6

Albert, N., Melau, M., Jensen, H., Emborg, C., Jepsen, J. R. M., Fagerlund, B., . . . Nordentoft, M. (2017). Five years of specialised early intervention versus two years of specialised early intervention followed by three years of standard treatment for patients with a first episode psychosis: randomised, superiority, parallel group trial in Denmark (OPUS II). *BMJ, 356*, i6681. doi:10.1136/bmj.i6681

Amminger, G. P., Harris, M. G., Conus, P., Lambert, M., Elkins, K. S., Yuen, H. P., & McGorry, P. D. (2006). Treated incidence of first-episode psychosis in the catchment area of EPPIC between 1997 and 2000. *Acta Psychiatrica Scandinavica, 114*(5), 337-345. doi:10.1111/j.1600-0447.2006.00790.x

Amsel, L. V., Hunter, N., Kim, S., Fodor, K. E., & Markowitz, J. C. (2012). Does a study focused on trauma encourage patients with psychotic symptoms to seek treatment? *Psychiatric Services, 63*(4), 386-389. doi:10.1176/appi.ps.201100251

Azrin, S. T., Goldstein, A. B., & Heinssen, R. K. (2015). Early intervention for psychosis: The Recovery After an Initial Schizophrenia Episode project. *Psychiatr. Ann., 45*(11), 548-553. doi:10.3928/00485713-20151103-05

Barrowclough, C., Haddock, G., Tarrier, N., Lewis, S. W., Moring, J., O'Brien, R., . . . McGovern, J. (2001). Randomized controlled trial of motivational interviewing, cognitive behavior therapy, and family intervention for patients with comorbid schizophrenia and substance use disorders. *American Journal of Psychiatry, 158*(10), 1706-1713. doi:10.1176/appi.ajp.158.10.1706

Baxter, R., Rabe-hesketh, S., & Parrott, J. (1999). Characteristics, needs and reoffending in a group of patients with schizophrenia formerly treated in medium security. *J. Forens. Psychiatr., 10*(1), 69-83. doi:10.1080/09585189908402140

Benedetti, G. (1974). What is psychotherapy of psychosis? *Psychotherapy and Psychosomatics, 24*(4-6), 327-336. doi:10.1159/000286754

Bernard, M., Jackson, C., & Jones, C. (2006). Written emotional disclosure following first‐episode psychosis: Effects on symptoms of post‐traumatic stress disorder. *British Journal of Clinical Psychology, 45*(3), 403-415. doi:10.1348/014466505x68933

Berry, K., Ford, S., Jellicoe-Jones, L., & Haddock, G. (2015). Trauma in relation to psychosis and hospital experiences: the role of past trauma and attachment. *Psychology and Psychotherapy, 88*(3), 227-239. doi:10.1111/papt.12035

Bertelsen, M., Jeppesen, P., Petersen, L., Thorup, A., Øhlenschlaeger, J., Le Quach, P., . . . Nordentoft, M. (2009). Course of illness in a sample of 265 patients with first-episode psychosis--five-year follow-up of the Danish OPUS trial. *Schizophrenia Research, 107*(2-3), 173-178. doi:10.1016/j.schres.2008.09.018

Bioque, M., Mezquida, G., Amoretti, S., García-Rizo, C., López-Ilundain, J. M., Diaz-Caneja, C. M., . . . Group, E. (2022). Clinical and treatment predictors of relapse during a three-year follow-up of a cohort of first episodes of schizophrenia. *Schizophrenia Research, 243*, 32-42. doi:10.1016/j.schres.2022.02.026

Bradley, A. C., Baker, A., & Lewin, T. J. (2007). Group intervention for coexisting psychosis and substance use disorders in rural Australia: outcomes over 3 years. *Australian and New Zealand Journal of Psychiatry, 41*(6), 501-508. doi:10.1080/00048670701332300

Breitborde, N. J. K., Bell, E. K., Dawley, D., Woolverton, C., Ceaser, A., Waters, A. C., . . . Harrison-Monroe, P. (2015). The Early Psychosis Intervention Center (EPICENTER): development and six-month outcomes of an American first-episode psychosis clinical service. *BMC Psychiatry, 15*(1), 266. doi:10.1186/s12888-015-0650-3

Brown, J. S., Stellrecht, N. E., Williams, F. M., Denoma, J. M., Wingate, L. R., Lima, E. N., . . . Joiner, T. E. (2005). A comparison of therapy alone versus therapy and medication in a community clinic. *Journal of Cognitive Psychotherapy, 19*(4), 309-316. doi:10.1891/jcop.2005.19.4.309

Burns, J. K., Jhazbhay, K., Esterhuizen, T., & Emsley, R. (2011). Exposure to trauma and the clinical presentation of first-episode psychosis in South Africa. *Journal of Psychiatric Research, 45*(2), 179-184. doi:10.1016/j.jpsychires.2010.05.014

Calhoun, P. S., Bosworth, H. B., Stechuchak, K. A., Strauss, J., & Butterfield, M. I. (2006). The impact of posttraumatic stress disorder on quality of life and health service utilization among veterans who have schizophrenia. *Journal of Traumatic Stress, 19*(3), 393-397. doi:10.1002/jts.20114

Calvert, C., Larkin, W., & Jellicoe-Jones, L. (2008). An exploration of the links between trauma and delusional ideation in secure services. *Behavioural and Cognitive Psychotherapy, 36*(5), 589-604. doi:10.1017/s1352465808004621

Chan, A. O., & Silove, D. (2000). Nosological implications of psychotic symptoms in patients with established posttraumatic stress disorder. *Australian and New Zealand Journal of Psychiatry, 34*(3), 522-525. doi:10.1080/j.1440-1614.2000.00723.x

Chiesa, M., Larsen-Paya, M., Martino, M., & Trinchieri, M. (2016). The relationship between childhood adversity, psychiatric disorder and clinical severity: results from a multi-centre study. *Psychoanal. Psychother., 30*(1), 79-95. doi:10.1080/02668734.2016.1145131

Clarke, A. M., McLaughlin, P., Staunton, J., Kerins, K., Power, B., Kearney, K., . . . Whitty, P. (2019). Retrospective study of first episode psychosis in the Dublin Southwest Mental Health Service: demographics, clinical profile and service evaluation of treatment. *Irish Journal of Psychological Medicine, 36*(4), 249-258. doi:10.1017/ipm.2017.46

Clarke, R., Kelly, R., & Hardy, A. (2022). A randomised multiple baseline case series of a novel imagery rescripting protocol for intrusive trauma memories in people with psychosis. *Journal of Behavior Therapy and Experimental Psychiatry, 75*(101699), 101699. doi:10.1016/j.jbtep.2021.101699

Coates, D., Wright, L., Moore, T., Pinnell, S., Merillo, C., & Howe, D. (2019). The psychiatric, psychosocial and physical health profile of young people with early psychosis: Data from an early psychosis intervention service. *Child Youth Serv., 40*(1), 93-115. doi:10.1080/0145935x.2018.1553613

Cohen, K., Edstrom, K., & Smith-Papke, L. (1995). Identifying early dropouts from a rehabilitation program for psychiatric outpatients. *Psychiatric Services, 46*(10), 1076-1078. doi:10.1176/ps.46.10.1076

Conus, P., Cotton, S., Schimmelmann, B. G., Berk, M., Daglas, R., McGorry, P. D., & Lambert, M. (2010). Pretreatment and outcome correlates of past sexual and physical trauma in 118 bipolar I disorder patients with a first episode of psychotic mania. *Bipolar Disord., 12*(3), 244-252. doi:10.1111/j.1399-5618.2010.00813.x

Conus, P., Cotton, S., Schimmelmann, B. G., McGorry, P. D., & Lambert, M. (2007). The First‐Episode Psychosis Outcome Study: premorbid and baseline characteristics of an epidemiological cohort of 661 first‐episode psychosis patients. *Early Intervention in A comparison of therapy alone versus therapy and medication in a community clinic, 1*(2), 191-200. doi:10.1111/j.1751-7893.2007.00026.x

Conus, P., Cotton, S., Schimmelmann, B. G., McGorry, P. D., & Lambert, M. (2017). Rates and predictors of 18-months remission in an epidemiological cohort of 661 patients with first-episode psychosis. *Social Psychiatry and Psychiatric Epidemiology, 52*(9), 1089-1099. doi:10.1007/s00127-017-1388-7

Cotton, S. M., Lambert, M., Schimmelmann, B. G., Foley, D. L., Morley, K. I., McGorry, P. D., & Conus, P. (2009). Gender differences in premorbid, entry, treatment, and outcome characteristics in a treated epidemiological sample of 661 patients with first episode psychosis. *Schizophrenia Research, 114*(1-3), 17-24. doi:10.1016/j.schres.2009.07.002

Coughlan, H., Healy, C., Ní Sheaghdha, Á., Murray, G., Humphries, N., Clarke, M., & Cannon, M. (2020). Early risk and protective factors and young adult outcomes in a longitudinal sample of young people with a history of psychotic-like experiences. *Early Intervention in Psychiatry, 14*(3), 307-320. doi:10.1111/eip.12855

Craig, T., Fennig, S., Tanenberg-Karant, M., & Bromet, E. J. (1999). Six-month clinical status as a predictor of 24-month clinical outcome in first-admission patients with schizophrenia. *Annals of Clinical Psychiatry, 11*(4), 197-203. doi:10.3109/10401239909147071

Craig, T. K. J., Johnson, S., McCrone, P., Afuwape, S., Hughes, E., Gournay, K., . . . Thornicroft, G. (2008). Integrated care for co-occurring disorders: psychiatric symptoms, social functioning, and service costs at 18 months. *Psychiatric Services, 59*(3), 276-282. doi:10.1176/ps.2008.59.3.276

Davis, M., Barad, M., Otto, M., & Southwick, S. (2006). Combining pharmacotherapy with cognitive behavioral therapy: traditional and new approaches. *Journal of Traumatic Stress, 19*(5), 571-581. doi:10.1002/jts.20149

de Bont, P. A. J. M., van der Vleugel, B. M., van den Berg, D. P. G., de Roos, C., Lokkerbol, J., Smit, F., . . . van Minnen, A. (2019). Health-economic benefits of treating trauma in psychosis. *European Journal of Psychotraumatology, 10*(1), 1565032. doi:10.1080/20008198.2018.1565032

Deblinger, E., Pollio, E., Runyon, M. K., & Steer, R. A. (2017). Improvements in personal resiliency among youth who have completed trauma-focused cognitive behavioral therapy: A preliminary examination. *Child Abuse and Neglect, 65*, 132-139. doi:10.1016/j.chiabu.2016.12.014

DeTore, N. R., Gottlieb, J. D., & Mueser, K. T. (2021). Prevalence and correlates of PTSD in first episode psychosis: Findings from the RAISE-ETP study. *Psychological Services, 18*(2), 147-153. doi:10.1037/ser0000380

Dryden-Mead, T., Nelson, B., & Bendall, S. (2022). "They may be confronting but they are good questions to be asking" young people's experiences of completing a trauma and PTSD screening tool in an early psychosis program. *Psychology and Psychotherapy, 95*(4), 1090-1107. doi:10.1111/papt.12420

Dubovsky, S. L., & Thomas, M. (1992). Psychotic depression: advances in conceptualization and treatment. *Hospital and Community Psychiatry, 43*(12), 1189-1198. doi:10.1176/ps.43.12.1189

Dyck, D. G., Short, R. A., Hendryx, M. S., Norell, D., Myers, M., Patterson, T., . . . McFarlane, W. R. (2000). Management of negative symptoms among patients with schizophrenia attending multiple-family groups. *Psychiatric Services, 51*(4), 513-519. doi:10.1176/appi.ps.51.4.513

Emsley, R., Oosthuizen, P., Niehaus, D., Koen, L., & Chiliza, B. (2007). Changing the course of schizophrenia - predictors of treatment outcome revisited. *South African Journal of Psychiatry, 13*(1), 5. doi:10.4102/sajpsychiatry.v13i1.4

Fallu, A., Kopala, L., Malla, A., & Thorpe, L. (1998). A young man with first-episode psychosis: case presentation and discussion. *The Canadian Journal of Psychiatry, 43 Suppl 1*(1_suppl), 4S-6S. doi:10.1177/07067437980430S102

Fleischhacker, W. W., Keet, I. P. M., Kahn, R. S., & Committee, E. S. (2005). The European First Episode Schizophrenia Trial (EUFEST): rationale and design of the trial. *Schizophrenia Research, 78*(2-3), 147-156. doi:10.1016/j.schres.2005.06.004

Folk, J. B., Tully, L. M., Blacker, D. M., Liles, B. D., Bolden, K. A., Tryon, V., . . . Niendam, T. A. (2019). Uncharted waters: Treating trauma symptoms in the context of early psychosis. *Journal of Clinical Medicine, 8*(9), 1456. doi:10.3390/jcm8091456

Freeman, D., Pugh, K., Vorontsova, N., Antley, A., & Slater, M. (2010). Testing the continuum of delusional beliefs: an experimental study using virtual reality. *Journal of Abnormal Psychology, 119*(1), 83-92. doi:10.1037/a0017514

Frueh, B. C., Buckley, T. C., Cusack, K. J., Kimble, M. O., Grubaugh, A. L., Turner, S. M., & Keane, T. M. (2004). Cognitive-behavioral treatment for PTSD among people with severe mental illness: a proposed treatment model. *Journal of Psychiatric Practice, 10*(1), 26-38. doi:10.1097/00131746-200401000-00004

Frueh, B. C., Cusack, K. J., Grubaugh, A. L., Sauvageot, J. A., & Wells, C. (2006). Clinicians' perspectives on cognitive-behavioral treatment for PTSD among persons with severe mental illness. *Psychiatric Services, 57*(7), 1027-1031. doi:10.1176/ps.2006.57.7.1027

Frueh, B. C., Grubaugh, A. L., Cusack, K. J., Kimble, M. O., Elhai, J. D., & Knapp, R. G. (2009). Exposure-based cognitive-behavioral treatment of PTSD in adults with schizophrenia or schizoaffective disorder: a pilot study. *Journal of Anxiety Disorders, 23*(5), 665-675. doi:10.1016/j.janxdis.2009.02.005

Fuller, P. R. (2010). Applications of trauma treatment for schizophrenia. *Journal of Aggression, Maltreatment & Trauma, 19*(4), 450-463. doi:10.1080/10926771003705114

Galletly, C., Van Hooff, M., & McFarlane, A. (2011). Psychotic symptoms in young adults exposed to childhood trauma--a 20 year follow-up study. *Schizophrenia Research, 127*(1-3), 76-82. doi:10.1016/j.schres.2010.12.010

Gibson, L. E., Reeves, L. E., Cooper, S., Olino, T. M., & Ellman, L. M. (2019). Traumatic life event exposure and psychotic-like experiences: A multiple mediation model of cognitive-based mechanisms. *Schizophrenia Research, 205*, 15-22. doi:10.1016/j.schres.2018.02.005

Gleeson, J., Wade, D., Castle, D., Gee, D., Crisp, K., Pearce, T., . . . McGorry, P. (2008). The EPISODE II trial of cognitive and family therapy for relapse prevention in early psychosis: Rationale and sample characteristics. *J. Ment. Health, 17*(1), 19-32. doi:10.1080/09638230701504999

Gottlieb, J. D., Mueser, K. T., Rosenberg, S. D., Xie, H., & Wolfe, R. S. (2011). Psychotic depression, posttraumatic stress disorder, and engagement in cognitive-behavioral therapy within an outpatient sample of adults with serious mental illness. *Comprehensive Psychiatry, 52*(1), 41-49. doi:10.1016/j.comppsych.2010.04.012

Grubaugh, A. L., Clapp, J. D., Frueh, B. C., Tuerk, P. W., Knapp, R. G., & Egede, L. E. (2016). Open trial of exposure therapy for PTSD among patients with severe and persistent mental illness. *Behaviour Research and Therapy, 78*, 1-12. doi:10.1016/j.brat.2015.12.006

Grubaugh, A. L., Veronee, K., Ellis, C., Brown, W., & Knapp, R. G. (2017). Feasibility and efficacy of prolonged exposure for PTSD among individuals with a psychotic spectrum disorder. *Frontiers in Psychology, 8*, 977. doi:10.3389/fpsyg.2017.00977

Haddock, G., Barrowclough, C., Tarrier, N., Moring, J., O'Brien, R., Schofield, N., . . . Lewis, S. (2003). Cognitive–behavioural therapy and motivational intervention for schizophrenia and substance misuse. *British Journal of Psychiatry, 183*(05), 418-426. doi:10.1192/bjp.183.5.418

Häfner, H., & an der Heiden, W. (1991). Evaluating effectiveness and cost of community care for schizophrenic patients. *Schizophrenia Bulletin, 17*(3), 441-451. doi:10.1093/schbul/17.3.441

Halpin, E., Kugathasan, V., Hulbert, C., Alvarez-Jimenez, M., & Bendall, S. (2016). Case formulation in young people with post-traumatic stress disorder and first-episode psychosis. *Journal of Clinical Medicine, 5*(11), 106. doi:10.3390/jcm5110106

Harris, A., Brennan, J., Anderson, J., Taylor, A., Sanbrook, M., Fitzgerald, D., . . . Gordon, E. (2005). Clinical profiles, scope and general findings of the Western Sydney First Episode Psychosis Project. *Australian and New Zealand Journal of Psychiatry, 39*(1-2), 36-43. doi:10.1111/j.1440-1614.2005.01517.x

Herz, M. I., Lamberti, J. S., Mintz, J., Scott, R., O'Dell, S. P., McCartan, L., & Nix, G. (2000). A program for relapse prevention in schizophrenia. *Archives of General Psychiatry, 57*(3), 277. doi:10.1001/archpsyc.57.3.277

Hirai, M., & Clum, G. A. (2005). An Internet-based self-change program for traumatic event related fear, distress, and maladaptive coping. *Journal of Traumatic Stress, 18*(6), 631-636. doi:10.1002/jts.20071

Hogg, B., Radua, J., Gardoki-Souto, I., Fontana-McNally, M., Lupo, W., Reinares, M., . . . Amann, B. L. (2024). EMDR therapy vs. supportive therapy as adjunctive treatment in trauma-exposed bipolar patients: A randomised controlled trial. *Spanish Journal of Psychiatry and Mental Health, 17*(4), 203-214. doi:10.1016/j.sjpmh.2023.11.005

Jackson, C., Bernard, M., & Birchwood, M. (2011). The efficacy of psychotherapy in reducing post-psychotic trauma. *Epidemiol. Psychiatr. Sci., 20*(2), 127-131. doi:10.1017/s2045796011000205

Jackson, C., Trower, P., Reid, I., Smith, J., Hall, M., Townend, M., . . . Birchwood, M. (2009). Improving psychological adjustment following a first episode of psychosis: a randomised controlled trial of cognitive therapy to reduce post psychotic trauma symptoms. *Behaviour Research and Therapy, 47*(6), 454-462. doi:10.1016/j.brat.2009.02.009

Kam, S. M., Singh, S. P., & Upthegrove, R. (2015). What needs to follow early intervention? Predictors of relapse and functional recovery following first-episode psychosis. *Early Intervention in Psychiatry, 9*(4), 279-283. doi:10.1111/eip.12099

Katsounari, I. (2015). Narrative exposure therapy for treating PTSD with psychotic features. *Clinical Case Studies, 14*(5), 342-356. doi:10.1177/1534650114559831

Katsuta, Y., Nishimatsu, Y., Saito, T., & Endo, S. (2003). Psychiatric intervention for Japanese nationals in New York. *Journal of Nippon Medical School, 70*(2), 141-150. doi:10.1272/jnms.70.141

Kevan, I. M., Gumley, A. I., & Coletta, V. (2007). Post‐traumatic stress disorder in a person with a diagnosis of schizophrenia: Examining the efficacy of psychological intervention using single N methodology. *Clinical Psychology & Psychotherapy, 14*(3), 229-243. doi:10.1002/cpp.534

Key, F. A., Craske, M. G., & Reno, R. M. (2003). Anxiety-based cognitive-behavioral therapy for paranoid beliefs. *Behavior Therapy, 34*(1), 97-115. doi:10.1016/s0005-7894(03)80024-2

Kilian, S., Asmal, L., Phahladira, L., Plessis, S. D., Luckhoff, H., Scheffler, F., . . . Emsley, R. (2020). The association between childhood trauma and treatment outcomes in schizophrenia spectrum disorders. *Psychiatry Research, 289*(113004), 113004. doi:10.1016/j.psychres.2020.113004

Kraan, T. C., Ising, H. K., Fokkema, M., Velthorst, E., van den Berg, D. P. G., Kerkhoven, M., . . . van der Gaag, M. (2017). The effect of childhood adversity on 4-year outcome in individuals at ultra high risk for psychosis in the Dutch Early Detection Intervention Evaluation (EDIE-NL) Trial. *Psychiatry Research, 247*, 55-62. doi:10.1016/j.psychres.2016.11.014

Kreis, I., Wold, K. F., Åsbø, G., Simonsen, C., Flaaten, C. B., Engen, M. J., . . . Melle, I. (2024). The relationship between visual hallucinations, functioning, and suicidality over the course of illness: a 10-year follow-up study in first-episode psychosis. *Schizophrenia (Heidelb.), 10*(1), 30. doi:10.1038/s41537-024-00450-8

Kuipers, E., Garety, P., Fowler, D., Freeman, D., Dunn, G., & Bebbington, P. (2006). Cognitive, emotional, and social processes in psychosis: refining cognitive behavioral therapy for persistent positive symptoms. *Schizophrenia Bulletin, 32 Suppl 1*(Supplement 1), S24-31. doi:10.1093/schbul/sbl014

Landgraf, S., Blumenauer, K., Osterheider, M., & Eisenbarth, H. (2013). A clinical and demographic comparison between a forensic and a general sample of female patients with schizophrenia. *Psychiatry Research, 210*(3), 1176-1183. doi:10.1016/j.psychres.2013.09.009

Leff, J. P. (1981). Prevention of relapse of schizophrenia by social and pharmacological treatments. *Bibliotheca Psychiatrica,* (160), 15-21. doi:10.1159/000392251

Long, M. E., Grubaugh, A. L., Elhai, J. D., Cusack, K. J., Knapp, R., & Frueh, B. C. (2010). Therapist fidelity with an exposure-based treatment of PTSD in adults with schizophrenia or schizoaffective disorder. *Journal of Clinical Psychology, 66*(4), 383-393. doi:10.1002/jclp.20657

Lutgens, D., Iyer, S., Joober, R., Brown, T. G., Norman, R., Latimer, E., . . . Malla, A. (2015). A five-year randomized parallel and blinded clinical trial of an extended specialized early intervention vs. regular care in the early phase of psychotic disorders: study protocol. *BMC Psychiatry, 15*(1), 22. doi:10.1186/s12888-015-0404-2

Malla, A., Norman, R., Bechard-Evans, L., Schmitz, N., Manchanda, R., & Cassidy, C. (2008). Factors influencing relapse during a 2-year follow-up of first-episode psychosis in a specialized early intervention service. *Psychological Medicine, 38*(11), 1585-1593. doi:10.1017/S0033291707002656

Marshall, C., Addington, J., Epstein, I., Liu, L., Deighton, S., & Zipursky, R. B. (2012). Treating young individuals at clinical high risk for psychosis. *Early Intervention in Psychiatry, 6*(1), 60-68. doi:10.1111/j.1751-7893.2011.00299.x

Mastroeni, A., Bellotti, C., Pellegrini, E., Galletti, F., Lai, E., & Falloon, I. R. H. (2005). Clinical and social outcomes five years after closing a mental hospital: a trial of cognitive behavioural interventions. *Clinical Practice and Epidemiology in Mental Health, 1*(1), 25. doi:10.1186/1745-0179-1-25

Mauritz, M., Goossens, P., Jongedijk, R., Vermeulen, H., & van Gaal, B. (2022). Investigating the efficacy and experiences with narrative exposure therapy in severe mentally ill patients with comorbid post-traumatic stress disorder receiving flexible assertive community treatment: A mixed methods study. *Frontiers in Psychiatry, 13*, 804491. doi:10.3389/fpsyt.2022.804491

McClellan, J., & Werry, J. (1994). Practice parameters for the assessment and treatment of children and adolescents with schizophrenia. *Journal of the American Academy of Child and Adolescent Psychiatry, 33*(5), 616-635. doi:10.1097/00004583-199406000-00002

McGowan, I. W., Fisher, N., Havens, J., & Proudlock, S. (2021). An evaluation of eye movement desensitization and reprocessing therapy delivered remotely during the Covid-19 pandemic. *BMC Psychiatry, 21*(1), 560. doi:10.1186/s12888-021-03571-x

Miller, D. J., Beck, N. C., & Fraps, C. (1984). Predicting rehospitalization at a community mental health center: A „double-crossed” validation. *Journal of Clinical Psychology, 40*(1), 35-39. doi:10.1002/1097-4679(198401)40:1<35::aid-jclp2270400106>3.0.co;2-8

Mitchell, S., Shannon, C., Mulholland, C., & Hanna, D. (2021). Reaching consensus on the principles of trauma-informed care in early intervention psychosis services: A Delphi study. *Early Intervention in Psychiatry, 15*(5), 1369-1375. doi:10.1111/eip.13068

Mkize, D. L. (2008). Post traumatic stress disorder symptoms in a psychiatric population not presenting with Trauma: a preliminary study. *African Journal of Psychiatry (Johannesbg.), 11*(1), 51-53. doi:10.4314/ajpsy.v11i1.30255

Møller, T., & Linaker, O. M. (2004). Symptoms and lifetime treatment experiences in psychotic patients with and without substance abuse. *Nordic Journal of Psychiatry, 58*(3), 237-242. doi:10.1080/08039480410006296

Møller, T., & Linaker, O. M. (2006). The long-term level of functioning in patients admitted for psychotic disorders with and without substance abuse. *Nordic Journal of Psychiatry, 60*(2), 121-125. doi:10.1080/08039480600583811

Morrison, A. P. (2004). The use of imagery in cognitive therapy for psychosis: a case example. *Memory, 12*(4), 517-524. doi:10.1080/09658210444000142

Morrison, A. P., Beck, A. T., Glentworth, D., Dunn, H., Reid, G. S., Larkin, W., & Williams, S. (2002). Imagery and psychotic symptoms: a preliminary investigation. *Behaviour Research and Therapy, 40*(9), 1053-1062. doi:10.1016/s0005-7967(01)00128-0

Mueser, K. T., & Butler, R. W. (1987). Auditory hallucinations in combat-related chronic posttraumatic stress disorder. *American Journal of Psychiatry, 144*(3), 299-302. doi:10.1176/ajp.144.3.299

Mueser, K. T., & Rosenberg, S. D. (2003). Treating the trauma of first episode psychosis: A PTSD perspective. *Journal of Mental Health, 12*(2), 103-108. doi:10.1080/096382300210000583371

Nigam, R., Schottenfeld, R., & Kosten, T. R. (1992). Treatment of dual diagnosis patients: a relapse prevention group approach. *Journal of Substance Abuse Treatment, 9*(4), 305-309. doi:10.1016/0740-5472(92)90023-h

Noordsy, D., Torrey, W., Mueser, K., Mead, S., O'Keefe, C., & Fox, L. (2002). Recovery from severe mental illness: an intrapersonal and functional outcome definition. *International Review of Psychiatry, 14*(4), 318-326. doi:10.1080/0954026021000016969

Norman, R. M. G., Manchanda, R., Harricharan, R., & Northcott, S. (2015). The course of negative symptoms over the first five years of treatment: Data from an early intervention program for psychosis. *Schizophrenia Research, 169*(1-3), 412-417. doi:10.1016/j.schres.2015.09.010

Norman, R. M. G., Manchanda, R., Malla, A. K., Windell, D., Harricharan, R., & Northcott, S. (2011). Symptom and functional outcomes for a 5 year early intervention program for psychoses. *Schizophrenia Research, 129*(2-3), 111-115. doi:10.1016/j.schres.2011.04.006

Oyewumi, L. K., & Savage, T. (2009). A partnership model of early intervention in psychosis programme--a Canadian experience. *Early Intervention in Psychiatry, 3*(3), 172-177. doi:10.1111/j.1751-7893.2009.00130.x

Patten, S. B., Williams, J. V., Petcu, R., & Oldfield, R. (2001). Delirium in psychiatric inpatients: a case-control study. *The Canadian Journal of Psychiatry, 46*(2), 162-166. doi:10.1177/070674370104600208

Peach, N., Alvarez-Jimenez, M., Cropper, S. J., Sun, P., & Bendall, S. (2019). Testing models of post-traumatic intrusions, trauma-related beliefs, hallucinations, and delusions in a first episode psychosis sample. *British Journal of Clinical Psychology, 58*(2), 154-172. doi:10.1111/bjc.12206

Pelizza, L., Leuci, E., Quattrone, E., Azzali, S., Paulillo, G., Pupo, S., . . . Menchetti, M. (2023). Borderline personality disorder vs. mood disorders: clinical comparisons in young people treated within an “Early Intervention” service for first episode psychosis. *The European Journal of Psychiatry, 37*(4), 100219. doi:10.1016/j.ejpsy.2023.07.002

Perkins, D. O., Gu, H., Weiden, P. J., McEvoy, J. P., Hamer, R. M., Lieberman, J. A., & Comparison of Atypicals in First Episode study, g. (2008). Predictors of treatment discontinuation and medication nonadherence in patients recovering from a first episode of schizophrenia, schizophreniform disorder, or schizoaffective disorder: a randomized, double-blind, flexible-dose, multicenter study. *Journal of Clinical Psychiatry, 69*(1), 106-113. doi:10.4088/jcp.v69n0114

Petti, E., Klaunig, M. J., Smith, M. E., Bridgwater, M. A., Roemer, C., Andorko, N. D., . . . Rakhshan Rouhakhtar, P. (2023). Mental health care utilization in individuals with high levels of psychosis-like experiences: Associations with race and potentially traumatic events. *Cultural Diversity & Ethnic Minority Psychology, 29*(3), 302-315. doi:10.1037/cdp0000500

Philips, B., Wennberg, P., Werbart, A., & Schubert, J. (2006). Young adults in psychoanalytic psychotherapy: patient characteristics and therapy outcome. *Psychology and Psychotherapy, 79*(Pt 1), 89-106. doi:10.1348/147608305X52649

Potkin, S. G., Alphs, L., Hsu, C., Krishnan, K. R. R., Anand, R., Young, F. K., . . . InterSe, P. T. S. G. (2003). Predicting suicidal risk in schizophrenic and schizoaffective patients in a prospective two-year trial. *Biological Psychiatry, 54*(4), 444-452. doi:10.1016/s0006-3223(03)00178-1

Puntis, S., Oke, J., & Lennox, B. (2018). Discharge pathways and relapse following treatment from early intervention in psychosis services. *BJPsych Open, 4*(5), 368-374. doi:10.1192/bjo.2018.50

Reiter, H., & Humphreys, L. (2021). Exposure, Relaxation, and Rescripting Therapy for trauma-related nightmares with psychiatric inpatients: A case series. *Clinical Case Studies, 20*(1), 3-21. doi:10.1177/1534650120953614

Riggs, S. E., Garrett, M., Arnold, K., Colon, E., Feldman, E. N., Huangthaisong, P., . . . Lee, E. (2016). Can frontline clinicians in public psychiatry settings provide effective psychotherapy for psychosis? *American Journal of Psychotherapy, 70*(3), 301-328. doi:10.1176/appi.psychotherapy.2016.70.3.301

Rowston, W. (2002). Early Psychosis intervention by a community mental health team. *Australas. Psychiatry, 10*(3), 236-241. doi:10.1046/j.1440-1665.2002.00455.x

Schandrin, A., Francey, S., Nguyen, L., Whitty, D., McGorry, P., Chanen, A. M., & O'Donoghue, B. (2023). Co-occurring first-episode psychosis and borderline personality pathology in an early intervention for psychosis cohort. *Early Intervention in Psychiatry, 17*(6), 588-596. doi:10.1111/eip.13352

Schimmelmann, B. G., Huber, C. G., Lambert, M., Cotton, S., McGorry, P. D., & Conus, P. (2008). Impact of duration of untreated psychosis on pre-treatment, baseline, and outcome characteristics in an epidemiological first-episode psychosis cohort. *Journal of Psychiatric Research, 42*(12), 982-990. doi:10.1016/j.jpsychires.2007.12.001

Shoval, G., Sever, J., Sher, L., Diller, R., Apter, A., Weizman, A., & Zalsman, G. (2006). Substance use, suicidality, and adolescent-onset schizophrenia: an Israeli 10-year retrospective study. *Journal of Child and Adolescent Psychopharmacology, 16*(6), 767-775. doi:10.1089/cap.2006.16.767

Slade, E. P., Gottlieb, J. D., Lu, W., Yanos, P. T., Rosenberg, S., Silverstein, S. M., . . . Mueser, K. T. (2017). Cost-effectiveness of a PTSD intervention tailored for individuals with severe mental illness. *Psychiatric Services, 68*(12), 1225-1231. doi:10.1176/appi.ps.201600474

Smith, H., Sawyer, D. A., & Way, B. B. (2002). Central New York Psychiatric Center: an approach to the treatment of co-occurring disorders in the New York State correctional mental health system. *Behavioral Sciences and the Law, 20*(5), 523-534. doi:10.1002/bsl.488

Swartz, M. S., Swanson, J. W., Hiday, V. A., Wagner, H. R., Burns, B. J., & Borum, R. (2001). A randomized controlled trial of outpatient commitment in North Carolina. *Psychiatric Services, 52*(3), 325-329. doi:10.1176/appi.ps.52.3.325

Tan, R., Gould, R. V., Combes, H., & Lehmann, S. (2014). Distress, trauma, and recovery: adjustment to first episode psychosis. *Psychology and Psychotherapy, 87*(1), 80-95. doi:10.1111/j.2044-8341.2012.02073.x

Thorup, A., Albert, N., Bertelsen, M., Petersen, L., Jeppesen, P., Le Quack, P., . . . Nordentoft, M. (2014). Gender differences in first-episode psychosis at 5-year follow-up--two different courses of disease? Results from the OPUS study at 5-year follow-up. *European Psychiatry, 29*(1), 44-51. doi:10.1016/j.eurpsy.2012.11.005

Tiffin, P., & Morrison, M. (2007). An early intervention in psychosis (eip) service for adolescents: The application of psychotherapeutic principles. *Psychoanalytic Psychotherapy, 21*(4), 267-283. doi:10.1080/02668730701698285

Trotta, A., Murray, R. M., David, A. S., Kolliakou, A., O'Connor, J., Di Forti, M., . . . Fisher, H. L. (2016). Impact of different childhood adversities on 1-year outcomes of psychotic disorder in the genetics and psychosis study. *Schizophrenia Bulletin, 42*(2), 464-475. doi:10.1093/schbul/sbv131

Turner, M. A., Boden, J. M., Smith-Hamel, C., & Mulder, R. T. (2009). Outcomes for 236 patients from a 2-year early intervention in psychosis service. *Acta Psychiatrica Scandinavica, 120*(2), 129-137. doi:10.1111/j.1600-0447.2009.01386.x

Uzenoff, S. R., Penn, D. L., Graham, K. A., Saade, S., Smith, B. B., & Perkins, D. O. (2012). Evaluation of a multi-element treatment center for early psychosis in the United States. *Social Psychiatry and Psychiatric Epidemiology, 47*(10), 1607-1615. doi:10.1007/s00127-011-0467-4

Valiente-Gómez, A., Moreno-Alcázar, A., Radua, J., Hogg, B., Blanco, L., Lupo, W., . . . Amann, B. L. (2019). A multicenter phase II rater-blinded randomized controlled trial to compare the effectiveness of Eye Movement Desensitization Reprocessing therapy vs. Treatment as usual in patients with substance use disorder and history of psychological trauma: A study design and protocol. *Frontiers in Psychiatry, 10*, 108. doi:10.3389/fpsyt.2019.00108

van den Berg, D. P., van der Vleugel, B. M., de Bont, P. A., Thijssen, G., de Roos, C., de Kleine, R., . . . van der Gaag, M. (2016). Exposing therapists to trauma-focused treatment in psychosis: effects on credibility, expected burden, and harm expectancies. *European Journal of Psychotraumatology, 7*(1), 31712. doi:10.3402/ejpt.v7.31712

van Nierop, M., Bak, M., de Graaf, R., Ten Have, M., van Dorsselaer, S., Genetic, R., . . . Outcome of Psychosis, G. I. (2015). The functional and clinical relevance of childhood trauma-related admixture of affective, anxious and psychosis symptoms. *Acta Psychiatrica Scandinavica, 133*(2), 91-101. doi:10.1111/acps.12437

Velthorst, E., Nelson, B., O'Connor, K., Mossaheb, N., de Haan, L., Bruxner, A., . . . Thompson, A. (2013). History of trauma and the association with baseline symptoms in an Ultra-High Risk for psychosis cohort. *Psychiatry Research, 210*(1), 75-81. doi:10.1016/j.psychres.2013.06.007

Verma, S., Poon, L. Y., Subramaniam, M., Abdin, E., & Chong, S. A. (2012). The Singapore Early Psychosis Intervention Programme (EPIP): A programme evaluation. *Asian Journal of Psychiatry, 5*(1), 63-67. doi:10.1016/j.ajp.2012.02.001

Vila-Badia, R., Del Cacho, N., Butjosa, A., Serra Arumí, C., Esteban Santjusto, M., Abella, M., . . . Usall, J. (2022). Prevalence and types of childhood trauma in first episode psychosis patients. Relation with clinical onset variables. *Journal of Psychiatric Research, 146*, 102-108. doi:10.1016/j.jpsychires.2021.12.033

Warren, J. M., Hanstock, T. L., Hunt, S. A., Halpin, S. A., Warner-Metzger, C. M., & Gurwitch, R. (2023). Utilizing parent-Child Interaction Therapy with trauma-Directed Interaction in a young male in out of home care who had experienced trauma. *Clinical Case Studies, 22*(3), 240-266. doi:10.1177/15346501221130532

Williams-Keeler, L., Milliken, H., & Jones, B. (1994). Psychosis as precipitating trauma for PTSD: a treatment strategy. *American Journal of Orthopsychiatry, 64*(3), 493-498. doi:10.1037/h0079543

Yanos, P. T., Vayshenker, B., Pleskach, P., & Mueser, K. T. (2016). Insight among people with severe mental illness, co-occurring PTSD and elevated psychotic symptoms: Correlates and relationship to treatment participation. *Comprehensive Psychiatry, 68*, 172-177. doi:10.1016/j.comppsych.2016.04.016

Yen, S., Shea, M. T., Battle, C. L., Johnson, D. M., Zlotnick, C., Dolan-Sewell, R., . . . McGlashan, T. H. (2002). Traumatic exposure and posttraumatic stress disorder in borderline, schizotypal, avoidant, and obsessive-compulsive personality disorders: Findings from the collaborative longitudinal personality disorders study. *Journal of Nervous and Mental Disease, 190*(8), 510-518. doi:10.1097/00005053-200208000-00003
